# Host sphingolipids support *Plasmodium berghei* liver stage development

**DOI:** 10.1128/mbio.01675-25

**Published:** 2025-07-21

**Authors:** Erin A. Schroeder, Isabel C. Colón, Porter E. Petruzziello, Emily R. Derbyshire

**Affiliations:** 1 Department of Molecular Genetics and Microbiology, Duke University School of Medicine 12277 , Durham, North Carolina, USA; 2 Department of Chemistry, Duke University 3065, Durham, North Carolina, USA; University of Geneva, Geneva, Switzerland

**Keywords:** *Plasmodium*, sphingolipid, host-parasite interactions, liver stage malaria, lipid trafficking, CERT1

## Abstract

During the asymptomatic liver stage*, Plasmodium* resides within a parasitophorous vacuole (PV) that protects the parasite from immune clearance while also restricting nutrient exchange with its host cell. Although it is known that *Plasmodium* must scavenge resources from its environment, the specific nutrients sequestered and the mechanisms for transporting them to the PV are poorly understood, particularly during the liver stage. In this study, we investigated the role of host lipids and discovered that sphingolipids are critical for both *Plasmodium berghei* liver stage development and invasion. Specifically, exogenous C16-ceramide enhanced parasite development and nuclear replication, while sphingomyelin in the host cell membrane was essential for parasite invasion. Live microscopy studies using NBD labeled sphingolipids further found that exogenous lipids are actively transported into the PV with sphingolipid scavenging occurring at all tested time points throughout the liver stage. This was, in part, supported by the host ceramide transporter, CERT1. CERT1 was enriched at the PV and genetic disruption significantly reduced both *P. berghei* load and ceramide trafficking into the PV. Finally, we identified proteins of the host salvage pathway as critical for the *Plasmodium* liver stage using chemical and genetic approaches. In particular, depletion of *CERS3* and *SPHK1* affected PV size and infection rate, but not invasion. Our findings enhance our understanding of host-parasite lipid interactions and may offer novel therapeutic targets to reduce disease burden.

**IMPORTANCE:**

*Plasmodium*, the causative agent of malaria, remains a significant global health challenge, placing approximately half the world’s population at risk of infection. Despite the existence of antimalarial treatments, the emergence of drug-resistant parasites highlights the urgent need to identify novel therapeutic targets. The *Plasmodium* liver stage represents a promising avenue for drug discovery as inhibiting parasite development would prevent both symptomatic disease and transmission to the mosquito vector. In this study, we examined the role of host sphingolipids and found that members perform distinct functions, supporting parasite invasion and/or development. We also identified several host proteins that influence *Plasmodium* liver stage viability and contribute to sphingolipid acquisition. In addition to their role in the liver stage, sphingolipids are known to be critical for the asexual and sexual blood stages, suggesting that targeting host sphingolipid metabolism could offer a novel multistage therapeutic strategy against malaria.

## INTRODUCTION

*Plasmodium,* the causative agent of malaria, continues to pose a significant global health challenge with an estimated 260 million cases and 600 thousand deaths in 2023 (1). Infection begins when the *Plasmodium* sporozoite is transmitted through the bite of an infected *Anopheles* mosquito to the human host. Sporozoites travel to the liver and traverse several hepatocytes before establishing productive infection by using a portion of the host cell membrane to form a parasitophorous vacuole (PV). During the liver stage, the parasite replicates within this PV to form thousands of merozoites capable of infecting and lysing red blood cells, ultimately causing the clinical symptoms of malaria (2). To support the massive expansion of parasites during the liver stage, host nutrients must be efficiently scavenged.

Lipids are among the essential nutrients required for *Plasmodium* liver stage development (3–5). They support parasite invasion, synthesis of parasite organelles, expansion of the parasitophorous vacuole membrane (PVM), and growth of the host cell as it accommodates the replicating parasite. While *Plasmodium* has the biosynthetic capacity to generate certain lipid species *de novo*, the proportions of lipids scavenged from the host cell vs those synthesized by the parasite are not fully understood. For example, the parasite’s type II fatty acid synthesis pathway is essential for *Plasmodium* late liver stage viability and merozoite formation (6–11). Conversely, *Plasmodium* must acquire cholesterol and phosphatidylcholine to maintain parasite load, both of which are known to be trafficked from the host cell (12–15). These findings indicate that *Plasmodium* is well adapted at synthesizing lipids and scavenging resources from their host cell.

A global lipidomic analysis of *Plasmodium berghei* infected hepatocytes has further highlighted significant alterations in infected cells during liver stage schizogony and early merozoite formation (15). Infected hepatocytes exhibit an enrichment in neutral lipids, phosphatidylcholine, sterols, and sphingolipids alongside a downregulation in all other phospholipids (15). To date, few mechanistic studies have linked these lipids to their function or acquisition. This includes host sphingolipids, whose role during the liver stage remains unknown despite compelling evidence of their importance during the asexual blood stage and gametogenesis (15–19). Sphingolipids are a structurally diverse class of lipids essential to all eukaryotes, playing key functions in maintaining membrane architecture, cell signaling, proliferation, and apoptosis. In hepatocytes, *de novo* sphingolipid synthesis occurs at the endoplasmic reticulum (ER), leading to the formation of ceramides, which are then trafficked to the Golgi for modifications including phosphorylation, glycosylation, and conversion to sphingomyelin (20). Sphingolipid trafficking is mediated by vesicles of the endomembrane system and specialized lipid transfer proteins, including the ceramide transporter 1 (CERT1) (21).

In this study, we screened a panel of lipids to understand their importance to the *Plasmodium* liver stage. We found the addition of exogenous C16-ceramide resulted in a dose-dependent increase in parasite load that correlated with an increase in both PV size and nuclear replication. Live microscopy studies with NBD-labeled sphingolipids further found that exogenous sphingolipids were actively trafficked into the PV, facilitated, in part, by the host ceramide transporter, CERT1. Not only were sphingolipids critical to parasite development, but we also observed that host cell membrane sphingomyelin was required for parasite invasion. Finally, we used chemical and genetic approaches to screen host sphingolipid metabolism and transportation pathways for their role in *Plasmodium* development. These assays uncovered a function for the host salvage pathway and additional lipid transfer proteins that may be leveraged by the parasite for survival during the liver stage. Together, this work highlights the essential nature of host sphingolipids for *Plasmodium* liver stage infection and reveals new potential host targets for therapeutic interventions against malaria.

## RESULTS

### Exogenous C16-ceramide enhances *P. berghei* liver stage development

**Fig 1:**
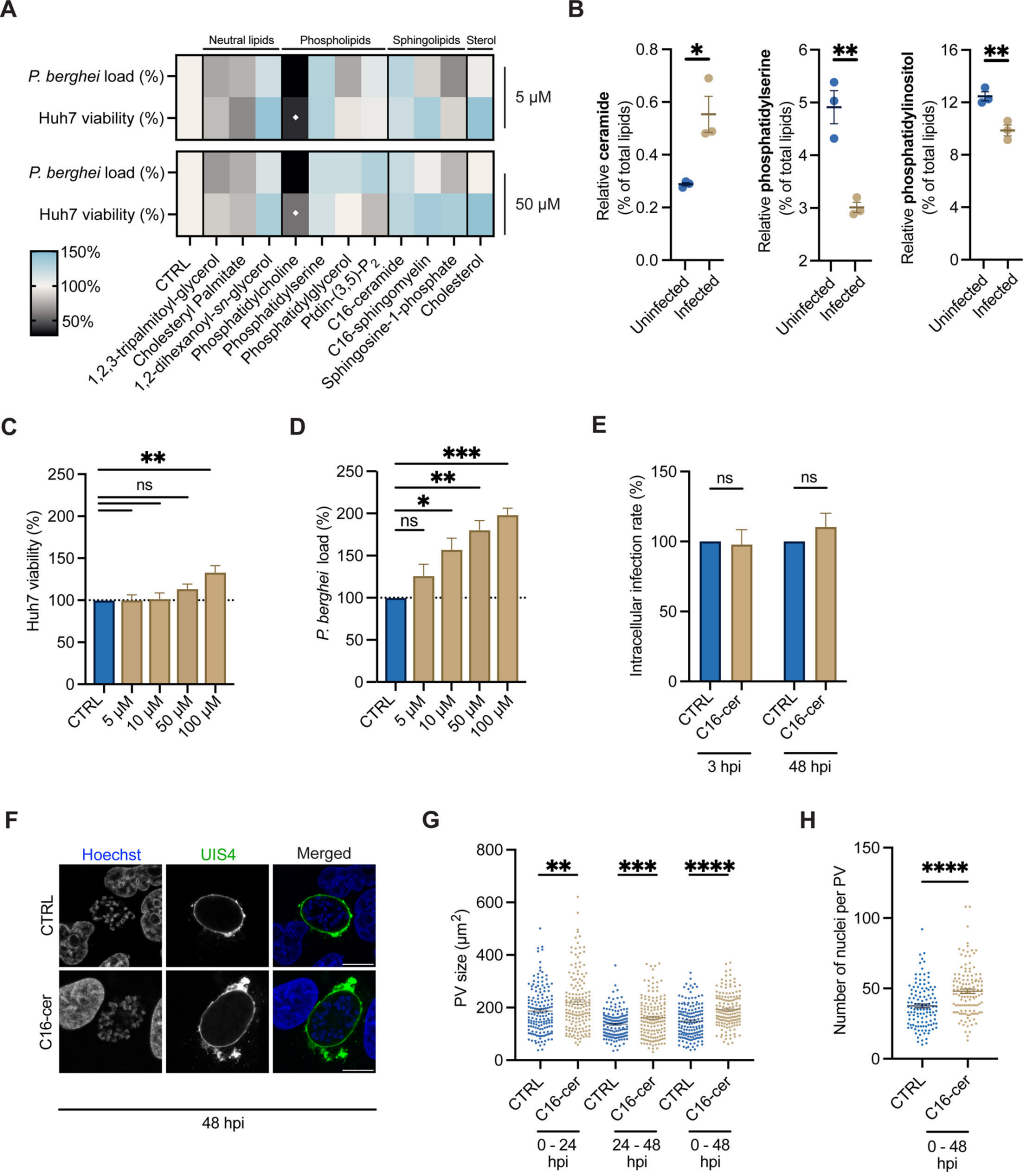
C16-ceramide promotes *P. berghei* liver stage development. (**A**) Huh7 cells infected with *P. berghei*-Luc were treated with a lipid panel at a final concentration of 5 and 50 µM at the time of infection. The relative parasite load was assessed at 48 hpi and compared to their respective solvent (CTRL = 100% viability). Data represent mean of four biological replicates. ♦ indicates Huh7 viability was less than 50% after lipid treatment. (**B**) The relative abundance of ceramide, phosphatidylcholine, and phosphatidylinositol was determined by quantitative shotgun lipidomics for uninfected and *P. berghei-*infected Huh7 cells at 35 hpi, performed by Itoe and colleagues (15). Data represent mean ± SEM. *n* = 3 biological replicates. *P-*values display unpaired *t*-test. *P < 0.05; **P < 0.01. (**C and D**) Huh7 cells were treated with increasing concentrations of C16-ceramide at the time of *P. berghei*-Luc infection. The relative (**C**) Huh7 viability and (**D**) *P. berghei* load were measured at 48 hpi and compared to cells treated with 1% ethanol (CTRL). Data represent mean ± SEM. *n* = 3 biological replicates. *P*-values display one-way ANOVA with Dunnett’s multiple comparison test for each condition to the CTRL. ns = non-significant; *P < 0.05; **P < 0.01; ***P < 0.005. Parasite load in panels **A** and **D** was assess with the luciferase reporter. (**E**) Huh7 cells were treated with 1% ethanol (CTRL) or 50 µM C16-ceramide at the time of *P. berghei* infection. At 3 and 48 hpi, cells were fixed, and the intracellular infection rate was evaluated by flow cytometry. Data represent mean ± SEM. *n* = 3 biological replicates. *P*-values display unpaired *t*-test. ns = non-significant. (**F**) Representative confocal immunofluorescence images of *P. berghei*-infected Huh7 cells treated with 1% ethanol (CTRL) or 50 µM C16-ceramide from 0 to 48 hpi. The PVM was stained with anti-UIS4 (green), and nuclei were stained with Hoechst (blue). Scale bars are 10 µm. (**G**) Huh7 cells infected with *P. berghei* were treated with 1% ethanol (CTRL) or 50 µM C16-ceramide from 0 to 24, 24 to 48, or 0 to 48 hpi. PV size was quantified by measuring the UIS4 area at 48 hpi. Data represent mean ± SEM. *n* = 3 biological replicates analyzing ≥50 PVs for each condition per biological replicate. *P-*values display unpaired *t*-test. **P < 0.01; ***P < 0.005; ****P < 0.001. (**H**) Huh7 cells infected with *P. berghei* were treated with 1% ethanol (CTRL) or 50 µM C16-ceramide from 0 to 48 hpi. Cells were stained with anti-UIS4 and Hoechst, Z-stacks of the PVs were acquired, and the number of parasite nuclei per PV was quantified. Data represent mean ± SEM. *n* = 3 biological replicates analyzing ≥40 PVs for each condition per biological replicate. *P-*value displays unpaired *t*-test. ****P < 0.001.

To understand the role of diverse host lipids during the *Plasmodium* liver stage, we investigated parasite viability following treatment with multiple exogenous lipids. First, we assessed a panel of lipids from four major classes including neutral lipids (1,2,3-tripalmitoyl-glycerol, cholesteryl palmitate, 1,2-dihexanoyl-*sn*-glycerol), phospholipids [phosphatidylcholine, phosphatidylserine, phosphatidylglycerol, PtdIns-(3,5)-P_2_], sphingolipids (C16-ceramide, C16-sphingomyelin, sphingosine-1-phosphate), and sterols (cholesterol). These lipids were selected based on their commercial availability, prior use in functional studies, and biophysical properties that support cellular experiments (22–27). Huh7 cells were treated with 5 and 50 µM of each lipid at the time of infection with *P. berghei* expressing luciferase (*P. berghei-*Luc). Cell viability and parasite load, as assessed with a fluorescence and luciferase reporter, respectively, were evaluated and compared to cells treated with their respective solvents at 48 hours post infection (hpi), which represents late liver stage development (Fig. 1A; Fig. S1A and B). Supplementation with phosphatidylcholine at both concentrations decreased Huh7 viability by greater than 40% from the control, and thus, parasite load could not be assessed. At a concentration of 5 µM, the only lipids that altered parasite load by more than 25% without affecting Huh7 viability were PtdINs-(3,5)-P_2_, which increased parasite load, and 1,2,3-tripalmitoyl glycerol, which decreased it. At the higher concentration of 50 µM, phosphatidylserine and C16-ceramide treatment increased parasite load above 100% when compared to the vehicle control. With this treatment, there was also a slight increase in Huh7 viability above 100%, but not all lipids that increased cell viability increased parasite load (e.g., C16-sphingomyelin). In contrast, many lipids that reduced Huh7 viability (e.g., cholesterol palmitate and sphingosine-1-phosphate) were associated with a decrease in parasite load. For functional studies, we sought to understand the role of lipids that increased parasite load (phosphatidylinositol, phosphatidylserine, and C16-ceramide). Among these lipids, we chose to focus on sphingolipids. This decision was based on a prior lipidomic analysis that showed elevated ceramide levels at 25 and 35 hpi when compared to uninfected cells, suggesting a potential functional role during infection (Fig. 1B and C; Fig. S1C) (15). In contrast, phosphatidylserine and phosphatidylinositol levels both decreased during *P. berghei* infection.

To further investigate ceramides, *P. berghei-*Luc-infected Huh7 cells were treated with different concentrations of C16-ceramide from 0 to 48 hpi before assessment of Huh7 viability and parasite load via its luciferase reporter. We observed a dose-dependent increase in parasite load that did not correlate with Huh7 viability (Fig. 1C and D). For subsequent studies, we used 50 µM C16-ceramide as it increased parasite load (>60*%, P* < 0.005) but did not significantly impact Huh7 viability (*P* = 0.33).

To elucidate the role of ceramide during liver stage infection, we employed a phenotypic approach involving flow cytometry and confocal microscopy. We assessed whether exogenous C16-ceramide specifically affects *P. berghei* invasion, survival (i.e., clearance from the host immune response), and/or development (i.e., PV size). Huh7 cells were infected with *P. berghei,* treated with C16-ceramide, and then fixed at 3 and 48 hpi. Infected cells were then stained with an antibody against UIS4, a PVM-resident protein, to identify infected cells by flow cytometry. At 3 and 48 hpi, we observed no change in the intracellular infection rate between control and C16-ceramide treated parasites (Fig. 1E; Fig. S2). Thus, C16-ceramide does not influence the parasite’s ability to invade hepatocytes or avoid clearance from its host cell. To then evaluate parasite development, *P. berghei*-infected Huh7 cells were treated with C16-ceramide from 0 to 24, 24 to 48, and 0 to 48 hpi. At 48 hpi, the PVM was labeled with an anti-UIS4 antibody, and the PV size was assessed by microscopy with >150 parasites analyzed per condition (Fig. 1F and G). A statistically significant increase in PV size was observed for each treatment window, with an average increase of 44.7 ± 7.3 µm^2^ (*P* < 0.0001) observed for C16-cermaide treatment from 0 to 48 hpi. To assert that exogenous ceramides promoted parasite development rather than simply expanding the PVM, the number of nuclei within the PV was quantified by Hoechst staining (Fig.1H and Fig. S3). Ceramide supplementation from 0 to 48 hpi significantly increased the number of parasite nuclei by 10 ± 2 (*P* < 0.0001) within a PV when compared to the vehicle control, indicating that ceramides promote liver stage PV growth and parasite nuclear replication.

Finally, we assessed the impact of ceramide fatty acid chain length on host cell viability and *P. berghei* load utilizing the luciferase reporter. *P. berghei*-Luc-infected Huh7 cells were treated with increasing concentrations of C6-, C12-, and C20-ceramides from 0 to 48 hpi. As previously described, high exogenous concentrations of C6- and C12-ceramides significantly reduced Huh7 viability and, thus, parasite load (Fig. S4) (28). In contrast, C20-ceramide corresponded with a >40% increase in *P. berghei* load similar to the observations for C16-ceramide. These data suggest that long-chain ceramides support parasite infection.

### Exogenous sphingolipids are trafficked into the PV of *P. berghei*

**Fig 2:**
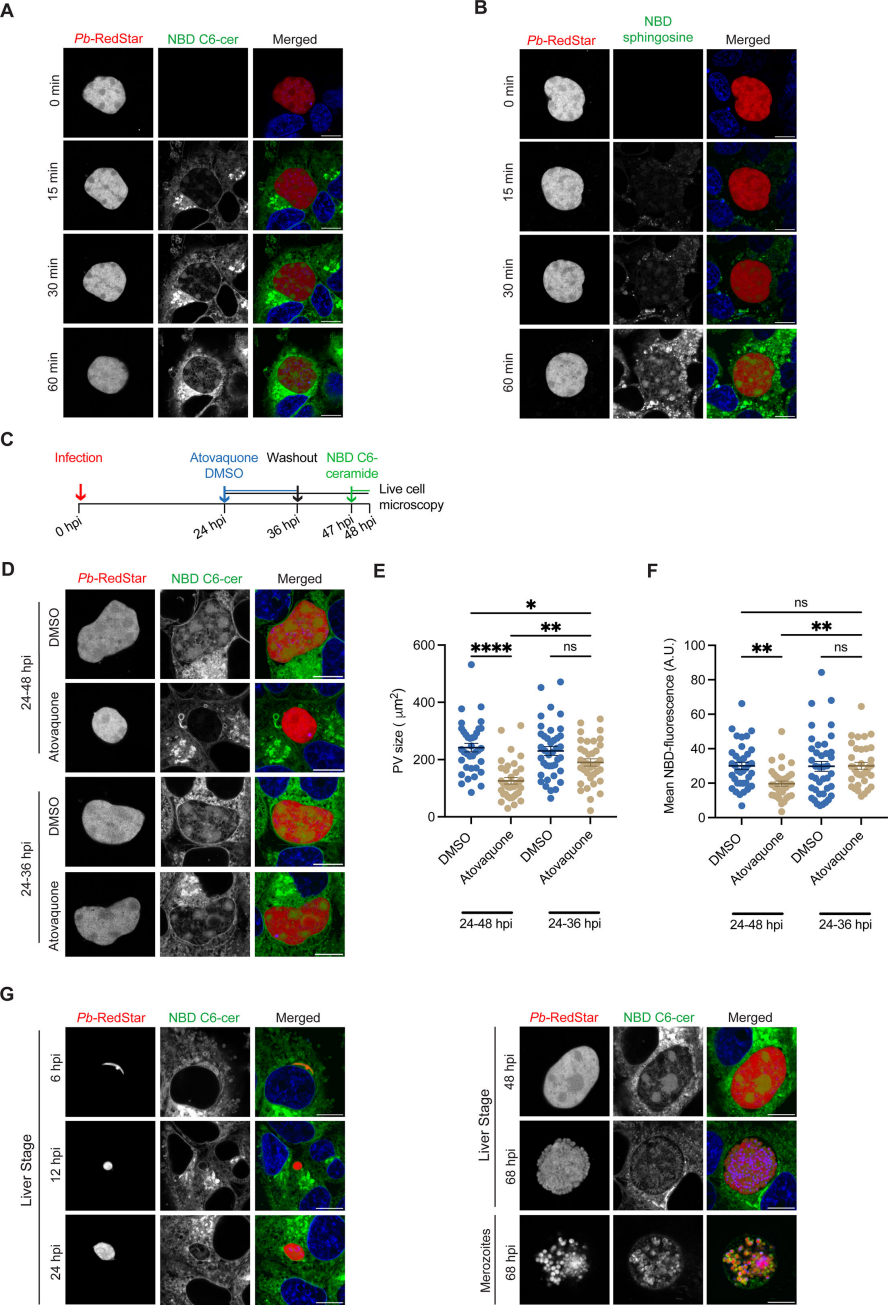
*P. berghei* traffics exogenous sphingolipids into the PV. (**A and B**) Live confocal fluorescent images of *P. berghei-*RedStar (red)-infected Huh7 cells at 48 hpi. Images represent a time lapse of the same parasite before and after the addition of (**A**) 5 µM NBD C6-ceramide (green) and (**B**) 5 µM NBD sphingosine (green) for the indicated incubation time. Nuclei were stained with Hoechst (blue). (**C–F**) Schematic representation of NBD C6-ceramide uptake experiments. Huh7 cells were infected with *P. berghei*-RedStar and then treated with DMSO or 10 nM atovaquone from 24 to 48 hpi or 24 to 36 hpi and then washed. Cells were subsequently supplemented with 5 µM NBD C6-ceramide for 1 h prior to live cell microscopy at 48 hpi. (**D**) Representative live confocal microscopy images displaying NBD C6-ceramide (green) uptake by *P. berghei*-RedStar (red)-infected Huh7 cells. Nuclei were stained with Hoechst (blue). Microscopy at 48 hpi was used to quantify the (**E**) PV size by measuring the RedStar area and (**F**) the mean NBD fluorescence value inside the PV. (**E and F**) Data represent mean ± SEM. *n* = 3 biological replicates analyzing ≥10 PVs for each condition per biological replicate. *P*-values display one-way ANOVA with Dunnett’s multiple comparison test. ns = non-significant; *P < 0.05; **P < 0.01; ****P < 0.001. (**G**) Representative live confocal microscopy images of *P. berghei-*RedStar (red)-infected Huh7 cells treated with 5 µM NBD C6-ceramide (green) for 1 h prior to microscopy at the indicated times post infection. Nuclei were stained with Hoechst (blue). (**A, B, D, G**) Scale bars are 10 µm.

We next aimed to explore whether liver stage *Plasmodium* parasites are capable of trafficking exogenous sphingolipids from their host cell. We first treated uninfected Huh7 cells with 5 µM NBD C6-ceramide, a commercially available and commonly used ceramide analog for fluorescence-based trafficking studies. Of note, cells were incubated for a shorter timeframe (<75 min) and at a lower lipid concentration where we observed no host or parasite toxicity (Fig. S5A and B)**.** As previously reported, our live microscopy studies found NBD C6-ceramide was internalized by host cells, accumulated at the Golgi complex, and dispersed throughout the host cytosol within 1 h (Fig. S5C) (29). We then infected Huh7 cells with *P. berghei* stably expressing RedStar (*P. berghei*-RedStar) and added 5 µM NBD C6-ceramide at 48 hpi. Within 15 min, we observed lipid dense regions inside of the PV that intensified over time around the parasite nuclei (Fig. 2A). Uninfected and *P. berghei*-RedStar-infected Huh7 cells were also probed with 5 µM NBD sphingosine, a structural component of ceramides. We again observed incorporation of the fluorescent lipid by host cells and rapid accumulation within the PV (Fig. 2B and Fig. S5D through F).

To determine whether *Plasmodium* actively acquires exogenous sphingolipids, *P. berghei*-RedStar-infected Huh7 cells were treated from 24 to 48 hpi with DMSO or 10 nM atovaquone, a mitochondrial *bc*_1_ complex inhibitor (Fig. 2C). We selected 10 nM atovaquone after testing various concentrations to identify one that reduced PV size without impacting PVM integrity as observed by confocal immunofluorescence microscopy (Fig. S6A). After atovaquone treatment, NBD C6-ceramide was introduced for 1 h prior to live cell microscopy at 48 hpi (Fig. 2D). Parasites treated with atovaquone had a significant reduction in PV size, indicating successful inhibition of the parasite metabolism (Fig. 2E). Furthermore, parasites treated with atovaquone had a significant reduction in the mean NBD fluorescent signal within the PV (*P* < 0.01) (Fig. 2F). Of note, no correlation was observed between PV size and NBD signal within the PV (Fig. S6B). To assess the reversibility of the atovaquone treatment, parasites were incubated with DMSO or 10 nM atovaquone from 24 to 36 hpi, washed to remove the inhibitor, and then allowed to develop until 48 hpi. Cells were then probed with NBD C6-ceramide for 1 h before live cell microscopy. The removal of atovaquone resulted in a significant increase in PV size compared to parasites continuously treated with atovaquone from 24 to 48 hpi (*P* < 0.01), as well as an increase in NBD C6-ceramide acquisition (*P* < 0.01) (Fig. 2C through F). These studies were replicated with NBD sphingosine, where a similar reduction and reversibility of the lipid uptake was observed (Fig. S7). Together, these data suggest that an active parasite metabolism is required for exogenous sphingolipid scavenging.

Finally, we hypothesized that sphingolipids are continuously acquired by liver stage parasites. To test this, *P. berghei*-RedStar-infected Huh7 cells were treated with 5 µM NBD C6-ceramide or NBD sphingosine for 1 h before live cell microscopy at 6, 12, 24, 48, and 68 hpi (Fig. 2G; Fig. S8). Fluorescent signal within the PV was observed at all tested time points and was seen around merozoites at 68 hpi, indicating that sphingolipids are continuously scavenged throughout parasite development and egress.

### Host CERT1 supports the *Plasmodium* liver stage and ceramide trafficking

To investigate possible mechanisms for ceramide trafficking to the PV, we focused our attention on CERT1. In mammalian cells, CERT1 can translocate a molecule of ceramide from the ER to the Golgi (21). Vijayan et al. also identified CERT1 in a genome-wide CRISPR screen as an essential host protein for *Plasmodium yoelii* liver stage development though it had no impact on invasion (30). We thus hypothesized that *Plasmodium* may exploit host CERT1 to acquire ceramides during the liver stage. To test this, we first investigated the localization of CERT1 during infection. Unfortunately, all commercially available antibodies against CERT1 cross-reacted with an unknown *Plasmodium* protein (data not shown). Therefore, we generated and overexpressed CERT1-V5 and CERT1-HA fusion proteins in HeLa cells. HeLa cells are well known to support liver stage *Plasmodium* development and have the advantage of a higher transfection efficiency when compared to Huh7 cells (31, 32). In uninfected cells, we observed that CERT1-V5 was enriched at the cis-Golgi, consistent with previous reports for the endogenous protein (Fig. S9A). In contrast, the CERT1-HA fusion protein appeared dispersed throughout the host cytosol and failed to associate at the Golgi (Fig. S9B). In subsequent studies, we employed CERT1-HA as a negative control due to this mislocalization.

**Fig 3:**
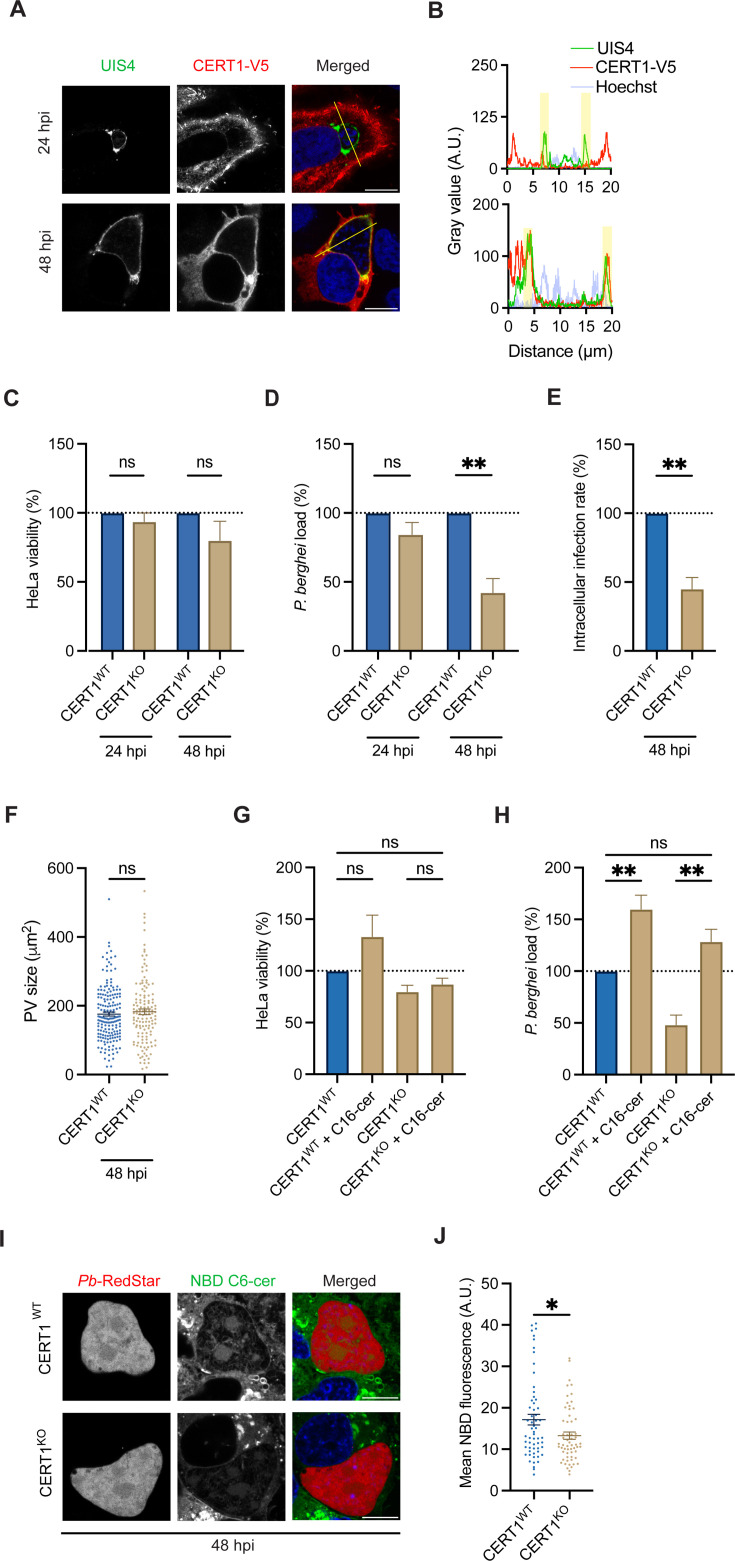
Host CERT1 supports *Plasmodium* liver stage and ceramide trafficking. (**A and B**) Representative confocal immunofluorescence images of HeLa cells overexpressing CERT1-V5 (red) and infected with *P. Berghei* at 24 and 48 hpi. the PVM was stained with anti-UIS4 (green), and nuclei were stained with Hoechst (blue). (**B**) Plot profile of the gray value for each fluorophore across the line (yellow). Relative (**C**) HeLa viability and (**D**) *P. berghei*-Luc load in CERT1^WT^ and CERT1^KO^ cells at 24 and 48 hpi. Data were normalized to CERT1^WT^ cells. Data represent mean ± SEM. *n* = 4 biological replicates. *P*-values display unpaired *t*-test. ns = non-significant; **P < 0.01. (**E and F**) CERT1^WT^ and CERT1^KO^ cells were infected with *P. berghei-*Luc, and the (**E**) relative intracellular infection rate and (**F**) PV size were analyzed at 48 hpi by quantifying the number of parasites per well and the UIS4 area by microscopy. Data represent mean ± SEM. *n* = 3 biological replicates. *P*-values display unpaired *t*-test. ns = non-significant; **P < 0.01. (**G and H**) CERT1^WT^ and CERT1^KO^ cells were infected with *P. berghei-*Luc and treated with 1% ethanol (CTRL) or 50 µM C16-ceramide. (**G**) Relative HeLa viability and (**H**) parasite load were assessed at 48 hpi. Data represent mean ± SEM. *n* = 4 biological replicates. *P*-values display unpaired *t*-test. ns = non-significant; **P < 0.01. (**I and J**) *P. berghei-*RedStar (red)-infected CERT1^WT^ and CERT1^KO^ cells were treated with 5 µM NBD C6-ceramide for 1 h prior to microscopy at 48 hpi. (**I**) Representative live confocal microscopy images displaying NBD C6-ceramide (green) and nuclei stained with Hoechst (blue). (**J**) Quantification of the mean NBD fluorescence value inside the PV. Data represent mean ± SEM. *n* = 4 biological replicates analyzing ≥15 PVs for each condition per biological replicate. *P-*value display unpaired *t*-test. *P < 0.05. (**A, I**) Scale bars are 10 µm.

Cells overexpressing CERT1-V5 were infected with *P. berghei* and fixed at 24 and 48 hpi. We assessed the relationship of CERT1-V5 and UIS4 localization using a plot profile and observed no notable enrichment of the host protein to the PVM at 24 hpi (Fig. 3A and B). However, at 48 hpi, CERT1-V5 and UIS4 showed an overlap, suggesting CERT1 was concentrating around the PVM (Fig. 3A and B; Fig. S9C). We also infected cells overexpressing CERT1-HA as a negative control and observed no association with the PVM (Fig. S9D). This indicates that not all overexpressed proteins associate at the PVM, a finding consistent with independent studies in the lab.

Next, we used CRISPR/Cas9 gene editing technology to disrupt *CERT1* expression in HeLa cells. gRNAs were designed to target exons 2 and 3, resulting in a clonal CERT1 disruption line (CERT1^KO^) (Fig. S10). CERT1 mRNA and protein levels were analyzed by qRT-PCR and western blot, respectively, revealing no detectable levels of CERT1 compared to wild-type HeLa (CERT1^WT^). CERT1^WT^ and CERT1^KO^ cells were then infected with *P. berghei-*Luc, and cell viability and parasite load (measured via luciferase reporter) were assessed at 24 and 48 hpi. No change in HeLa viability or parasite load was observed at 24 hpi (Fig. 3C and D). At 48 hpi, no change in HeLa viability was observed (*P* = 0.20), but there was a 58% ± 10% reduction in parasite load (*P* < 0.005). For a phenotypic investigation, we next assessed parasite infection and development at 48 hpi via microscopy. We observed an approximate 55% decrease in the number of infected cells but no change in the PV size (Fig. 3E and F). These observations suggest that parasites reliant on CERT1 for ceramide acquisition are cleared by the host cell, whereas those that find alternative mechanisms continue to develop.

As the primary function of CERT1 is ceramide transport, we sought to test if exogenous ceramide could rescue the effect of *CERT1* disruption on parasite load, as measured by a luciferase reporter. CERT1^WT^ and CERT1^KO^ cells were treated with 50 µM C16-ceramide concurrent with *P. berghei*-Luc infection. At 48 hpi, C16-ceramide had no significant impact on HeLa CERT1^WT^ viability (*P* = 0.16) but increased *P. berghei* load by 60% ± 14% (*P* < 0.005), consistent with observations in *P. berghei*-infected Huh7 cells (Fig. 3G and H). Strikingly, the addition of C16-ceramide in CERT^KO^ cells rescued parasite load to CERT1^WT^ levels. In contrast, the addition of the related sphingolipid, C16-sphingomyelin, had no impact on parasite load in CERT1^KO^ cells (*P* = 0.09), and thus, the rescue phenotype is specific to C16-ceramide (Fig. S11A and B).

Given the rescue of parasite load with exogenous C16-ceramide and the localization of CERT1 to the PVM, we hypothesized CERT1 could support sphingolipid trafficking to the PV. To test this, CERT1^WT^ and CERT1^KO^ cells were treated with 5 µM NBD C6-ceramide and examined with live cell microscopy over 1 h. No change in host cell acquisition or enrichment of NBD C6-ceramide at the host Golgi was observed after *CERT1* disruption (Fig. S11C). CERT1^WT^ and CERT1^KO^ cells were then infected with *P. berghei-*RedStar and treated with 5 µM NBD C6-ceramide for 1 h prior to live cell microscopy at 48 hpi (Fig. 3I). The mean NBD fluorescence inside the PV of CERT1^KO^ cells was significantly reduced compared to CERT1^WT^ cells (*P* < 0.05), suggesting that CERT1 contributes to ceramide trafficking to the PV (Fig. 3J).

### Host cell sphingomyelin is required for *P. berghei* invasion

**Fig 4:**
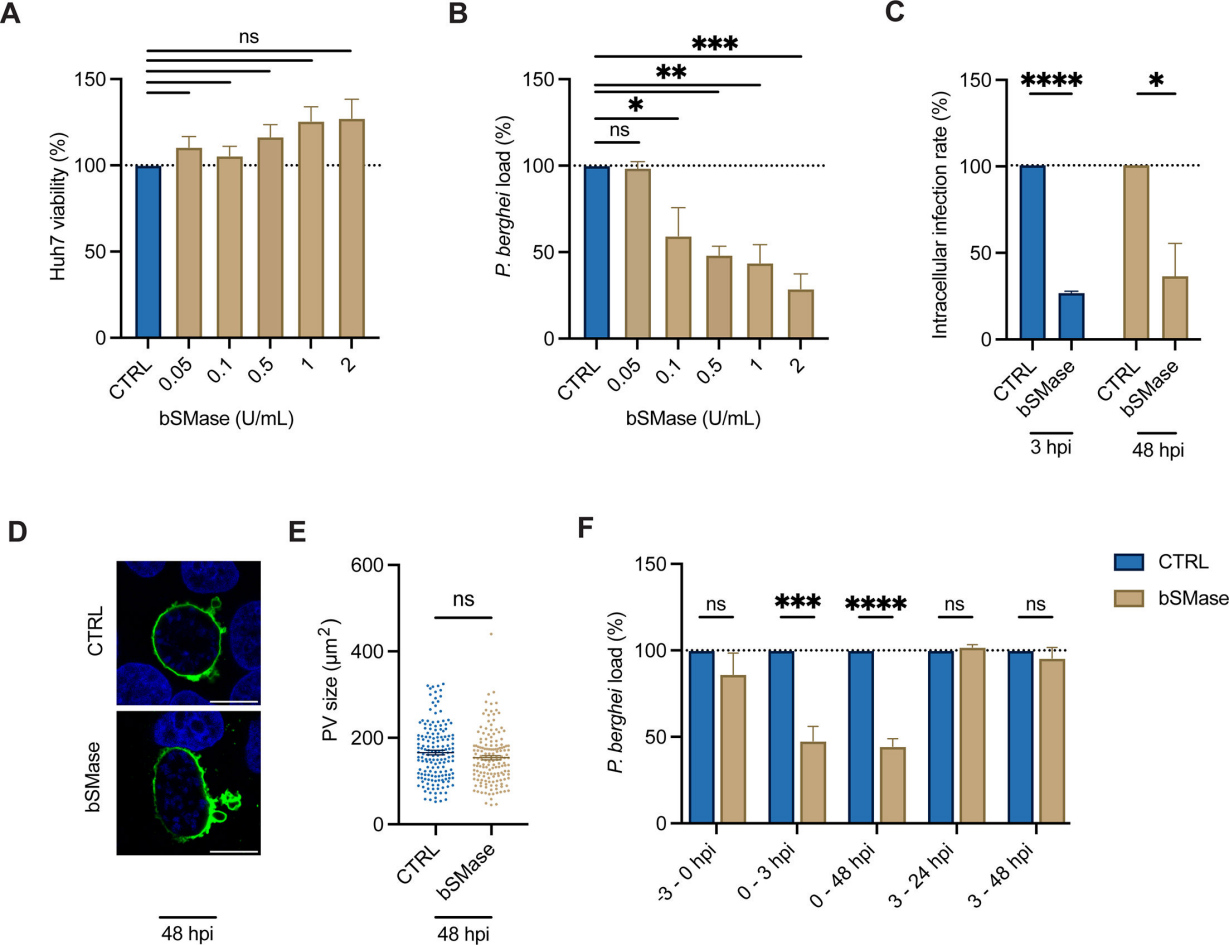
Host cell membrane sphingomyelin is required for *P. berghei* invasion. (A and B) Huh7 cells infected with *P. berghei*-Luc were treated with 25% glycerol in PBS (CTRL) or increasing concentrations of bSMase at the time of infection. At 48 hpi, the relative (**A**) Huh7 viability and (**B**) *P. berghei* load were assessed and normalized to the CTRL. Data represent mean ± SEM. *n* = 3 biological replicates. *P*-values display one-way ANOVA with Dunnett’s multiple comparison test for each condition compared to the CTRL. ns = non-significant; *P < 0.05; **P < 0.01; ***P < 0.005. (**C**) Huh7 cells infected with *P. berghei-*Luc were treated with 25% glycerol in PBS (CTRL) or 2 U/mL bSMase at the time of infection. At 3 and 48 hpi, cells were fixed, and the relative intracellular infection rate was quantified by flow cytometry. Data represent mean ± SEM. *n* = 3 biological replicates. *P*-values display unpaired *t*-test. *P < 0.05; ****P < 0.001. (**D and E**) Huh7 cells infected with *P. berghei*-Luc were treated with 25% glycerol in PBS (CTRL) or 0.5 U/mL bSMase at the time of infection and allowed to develop for 48 hpi. (**D**) Representative confocal immunofluorescence images displaying PVM stained with anti-UIS4 (green) and nuclei stained with Hoechst (blue). Scale bars are 10 µm. (**E**) The PV size was quantified by measuring the UIS4 area by microscopy. Data represent mean ± SEM. *n* = 3 biological replicates analyzing ≥50 PVs for each condition per biological replicate. *P-*values display unpaired *t*-test. ns = non-significant. (**F**) Huh7 cells infected with *P. berghei*-Luc were treated with 25% glycerol in PBS (CTRL) or 0.5 U/mL bSMase for indicated timepoints. At 48 hpi, *P. berghei* load was analyzed and normalized to cells treated with the CTRL. Data represent mean ± SEM. *n* = 3-4 biological replicates. *P*-values display unpaired *t*-test. ns = non-significant; ***P < 0.005; ****P < 0.001.

After noting the impact of sphingolipids on *Plasmodium* development, we questioned whether lipids on the host cell membrane could be important for parasite invasion. To investigate this, we focused on sphingomyelin as it is the predominant sphingolipid species of the host cell membrane (33, 34). We used bacterial sphingomyelinase (bSMase), an enzyme that selectively breaks down sphingomyelin into ceramide on the outer host cell membrane, to reduce sphingomyelin and thereby elevate ceramide levels (35). To test the effectiveness of bSMase in our system, Huh7 cells were treated with 0.5 U/mL bSMase for 2 h before fixation and staining with an anti-ceramide antibody. As anticipated, bSMase treatment significantly increased ceramide levels (Fig. S12A and B). Huh7 cells were then treated with increasing concentrations of bSMase, infected with *P. berghei*-Luc, and evaluated at 48 hpi for changes in host cell viability and parasite load (via luciferase signal). While no host cell cytotoxicity was observed at any of the tested enzyme concentrations, there was a dose-dependent decrease in parasite load (Fig. 4A and B). To ensure that bSMase was not inducing parasite death before invasion, sporozoites were pretreated with 2 U/mL bSMase at varying temperatures and incubation times. Pretreated sporozoites were washed to remove bSMase or used directly for infection to yield a final concentration of 0.1 U/mL (20-fold dilution). Pretreated and washed parasites exhibited no change in *P. berghei* load at 48 hpi under the three different experimental conditions. In contrast, a greater than 50% reduction in parasite load was observed when bSMase was not removed (Fig. S12C). These findings suggest that bSMase acts on the host cell to inhibit *Plasmodium* load.

We hypothesized that sphingomyelin in the host cell membrane facilitates parasite invasion. To specifically evaluate the impact on invasion, Huh7 cells were treated with 2.0 U/mL bSMase at the time of *P. berghei* infection and fixed at 3 hpi. As a positive control, *P. berghei*-infected Huh7 cells were treated with 0.5 µM cytochalasin D, an actin polymerization inhibitor that impedes *Plasmodium* traversal and, thus, prevents invasion (36). Fixed cells were stained with anti-UIS4 and analyzed by flow cytometry. We found that cytochalasin D and bSMase treatment decreased the relative intracellular infection rate at 3 hpi by 80% ± 3.8% (*P* < 0.0001) and 74% ± 2.1% (*P* < 0.0001), respectively (Fig. 4C; Fig. S13). Given that bSMase treatment significantly reduced invasion, we considered whether this effect was due to an increase in ceramide or depletion of sphingomyelin. However, as we previously demonstrated that the addition of C16-ceramide had no impact on invasion (Fig. 1E), these findings suggest that sphingomyelin facilitates parasite invasion.

We next assessed the fitness of parasites that successfully invaded host cells during bSMase treatment to determine if host cell membrane sphingomyelin was important for development. Cells were treated with 2.0 U/mL bSMase from 0 to 48 hpi, fixed at 48 hpi, and analyzed by flow cytometry. We observed a similar infection rate at 3 and 48 hpi, indicating the rate of host elimination was not impacted by sphingomyelin depletion or ceramide elevation at the host cell membrane (Fig. 4C). We next examined the PV size by immunofluorescent confocal microscopy at 48 hpi and found no difference between bSMase and control treated parasites (*P* = 0.09) indicating that cell membrane sphingomyelin is not critical for parasite development after invasion (Fig. 4D and E). To validate these results, we treated infected cells with 0.5 U/mL bSMase at various times before and after infection. Parasite load, as assessed by the luciferase reporter, was only reduced when cell membrane sphingomyelin was depleted between 0 and 3 hpi when parasites are undergoing invasion and traversal (Fig. 4F).

### Multiple host sphingolipid pathways support *Plasmodium* liver stage

**Fig 5:**
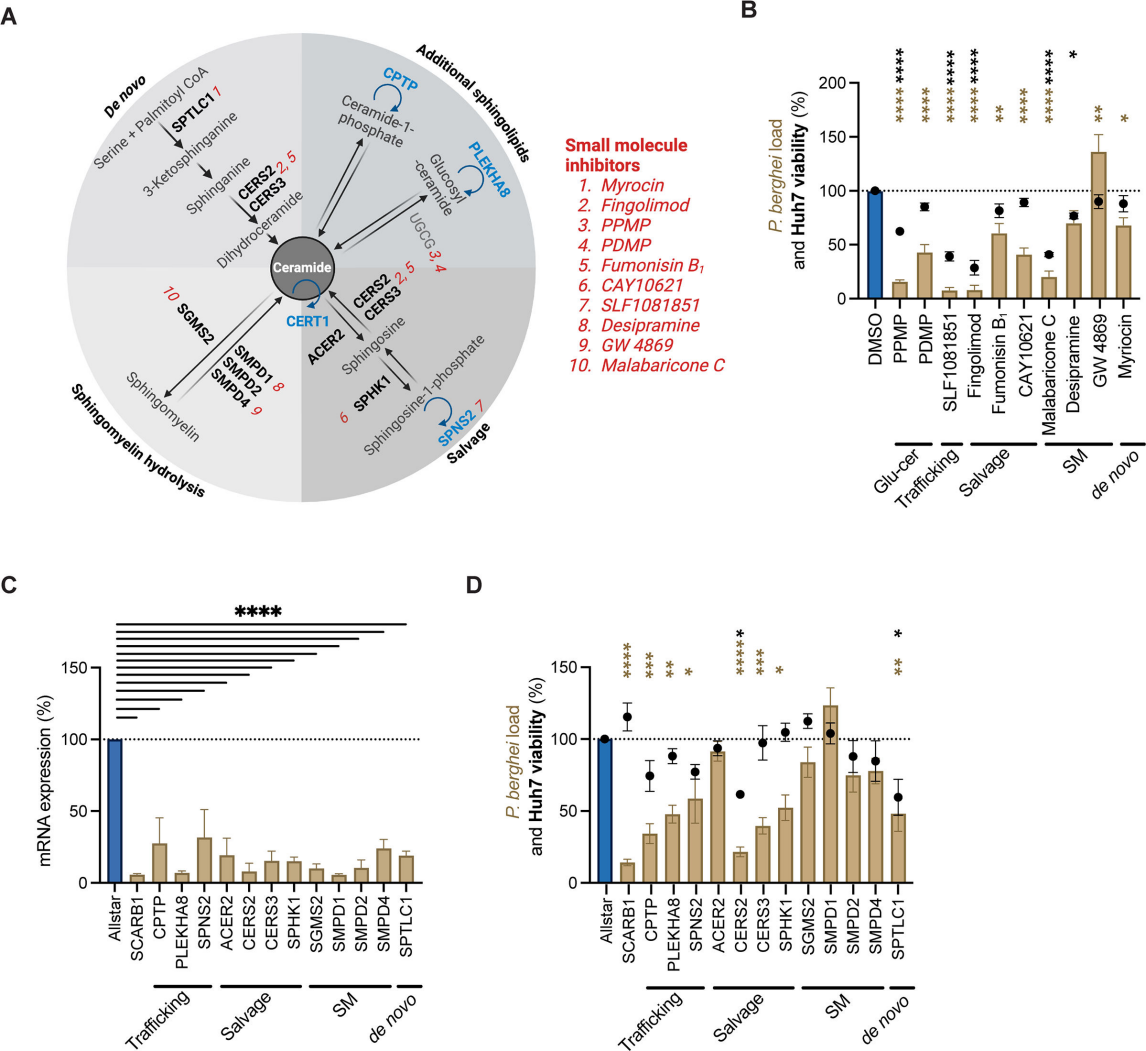
Host sphingolipid pathways are required during the *P. berghei* liver stage. (**A**) Sphingolipid pathways involved in ceramide metabolism including *de novo* synthesis, sphingomyelin hydrolysis, salvage, and additional complex sphingolipids. Host genes targeted by siRNAs (bolded) and small molecule inhibitors (red numbers) were investigated to identify host pathways critical to *Plasmodium.* Lipid transport proteins are denoted in blue. (**B**) *P. berghei*-Luc infected Huh7 cells were treated with small molecules at a final concentration of 10 µM at the time of infection. Huh7 viability (black circles) and *P. berghei* load (bars) were assessed at 48 hpi. Data represent mean ± SEM. *n* = 4 biological replicates. *P*-values display one-way ANOVA with Dunnett’s multiple comparison test for each condition compared to DMSO. *P < 0.05; **P < 0.01; ****P < 0.001. (**C and D**) Huh7 cells were reverse transfected (25 nM) with a non-targeting scramble control (Allstar, blue), a positive control for decreased parasite load (SCARB1), or two pooled siRNAs targeting host genes of interest (brown) for 48 h prior to infection with *P. berghei*-Luc. (**C**) The relative mRNA levels for genes of interest were assessed by qRT-PCR. (**D**) Huh7 viability (black circles) and *P. berghei* load (bars) were assessed at 48 hpi. Data represent mean ± SEM. *n* = 3 biological replicates. *P*-values display one-way ANOVA with Dunnett’s multiple comparison test for each condition compared to siAllstar. *P < 0.05; **P < 0.01; ***P < 0.005; ****P < 0.001. Bars without black (Huh7 viability) or brown (*P. berghei* load) asterisks had *P-*values > 0.05 and were not considered to differ significantly from the control.

After demonstrating the role of host CERT1 in ceramide acquisition, we aimed to explore if *Plasmodium* exploits other host sphingolipid pathways (37). We specifically investigated host *de novo* synthesis (*SPTLC1, CERS2,* and *CERS3*), recycling of complex sphingolipids (*UGCG*), salvage (*ACER2*, *CERS2*, *CERS3*, and *SPHK1*), sphingomyelin hydrolysis (*SGMS2*, *SMPD1*, *SMPD2*, and *SMPD4*), and lipid transfers proteins (*SPNS2*, *PLEKHA8*, and *CPTP*). To do this, we combined pathway-targeting small molecule inhibitors and a siRNA collection to assess the role of host genes on parasite load (Fig. 5A).

Huh7 cells were treated with 10 small molecule inhibitors at a final concentration of 1 and 10 µM and infected with *P. berghei*-Luc. Cell viability and parasite load (via luciferase signal) were assessed at 48 hpi, and statistically significant inhibition was identified using a one-way ANOVA with Dunnett’s multiple comparison test. *P*-values exceeding 0.05 were not considered to differ significantly from the control. At a concentration of 1 µM, only desipramine, an inhibitor of SMPD1, significantly reduced parasite viability (Fig. S14) (38). At 10 µM, five compounds exhibited a significant decrease in host cell viability and, thus, could not be evaluated (Fig. 5B). Conversely, PDMP (targets UGCG), fumonisin B_1_ (targets CERS2 and CERS3), CAY10621 (targets SPHK1), and myriocin (targets SPTLC1) all decreased parasite load without a significant impact on host cell viability (39–42). While the primary target for each inhibitor is indicated, off-target effects are known liabilities of small molecules. Fumonisin B_1_, for example, is a broad-spectrum inhibitor of ceramide synthases and may have impacts on other ceramide synthases (43). To address this concern, siRNAs were employed to deplete the expression of specific host genes.

Huh7 cells were reverse transfected for 48 h with 25 nM of two pooled siRNAs against different host sphingolipid targets, along with a non-targeting scramble control (Allstar), and an siRNA for a known host factor critical to *Plasmodium* liver stage invasion and development (SCARB1) (44, 45). By qRT-PCR, we found that there was efficient mRNA knockdown of each target (<40% expression) (Fig. 5C). Reverse transfected cells were then infected with *P. berghei*-Luc and assessed for cell viability and parasite load at 48 hpi, where statistically significant inhibition was identified using a one-way ANOVA with Dunnett’s multiple comparison test. Again, *P*-values exceeding 0.05 were not considered to differ significantly from the control. We found that knockdown of genes involved in the host sphingomyelin pathway did not impact *P. berghei* load, as measured with the luciferase reporter (Fig. 5D). In contrast, gene reduction of lipid transfer proteins (CPTP, PLEKHA8, and SPNS2) or members of the salvage pathway (CERS3 and SPHK1) decreased parasite load without significantly impacting Huh7 viability. Genetic depletion of SPTLC1, the first enzyme in the host *de novo* sphingolipid pathway, and CERS2, a ceramide synthase, both decreased host cell viability and, thus, could not be considered in this analysis.

### The host salvage pathway is critical for *P. berghei* development

**Fig 6:**
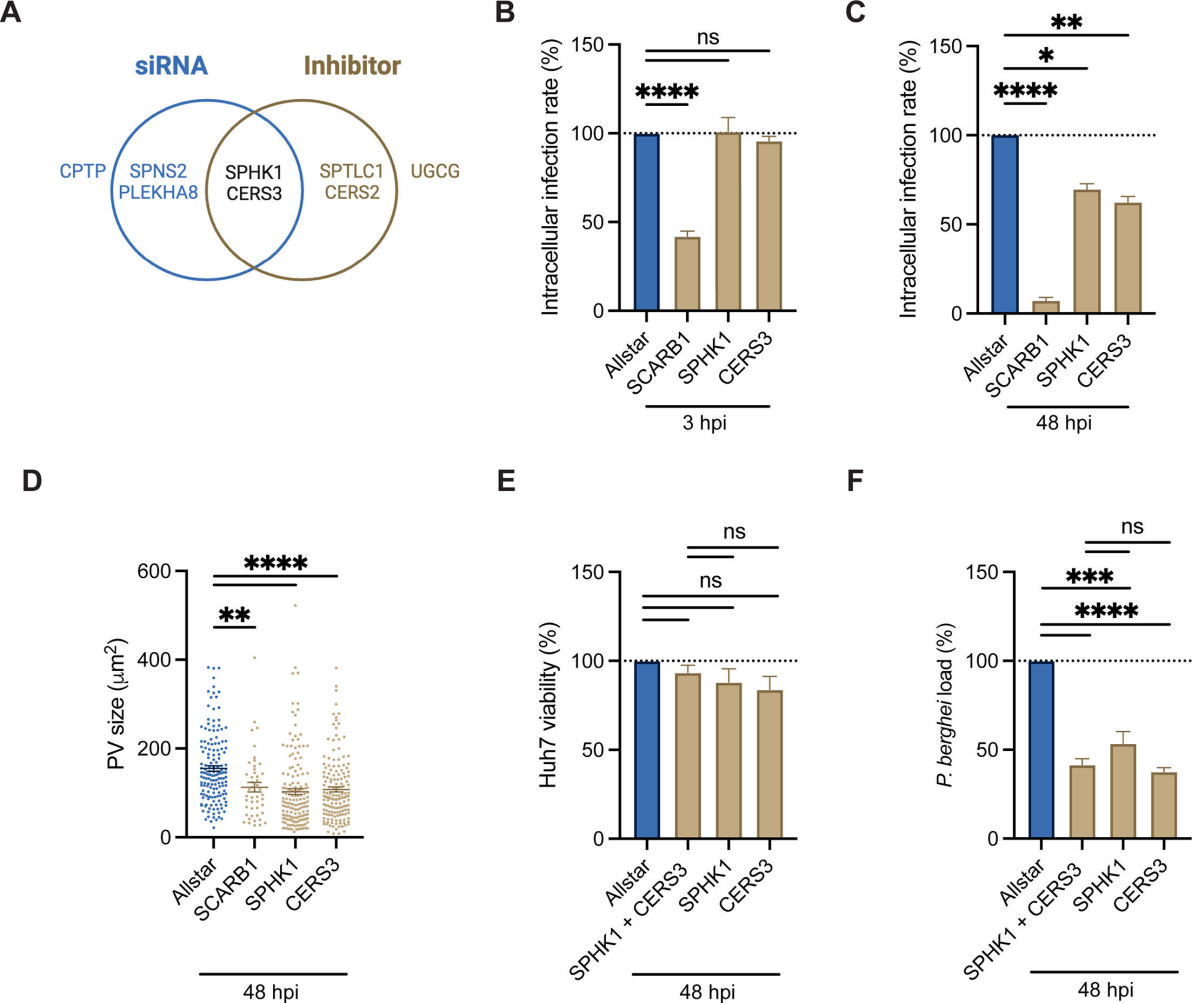
The host salvage pathway is critical for *P. berghei* development. (**A**) Venn diagram demonstrating overlap in targets from siRNA (blue circle) and small molecule (brown circle) studies, highlighting SPHK1 and CERS3 as potential host factors required for *P. berghei* development. (**B–D**) Huh7 cells were reverse transfected (25 nM) with a non-targeting control (Allstar, blue), a positive control (SCARB1), or two pooled siRNAs targeting *CERS3* or *SPHK1* for 48 h prior to infection with *P. berghei*-Luc. (**B**) At 3 hpi, cells were fixed, and the rate of hepatocyte invasion was assessed by flow cytometry. Data represent mean ± SEM. *n* = 3 biological replicates. *P*-values display unpaired *t*-test. ns = non-significant; ****P < 0.001. (**C**) At 48 hpi, the relative intracellular infection rate was analyzed by quantifying the number of parasites per well by confocal immunofluorescence microscopy. Data represent mean ± SEM. *n* = 3 biological replicates. *P*-values display unpaired *t*-test. *P < 0.05; **P < 0.01; ****P < 0.001. (**D**) The PV size was quantified by measuring the UIS4 area by confocal immunofluorescence microscopy at 48 hpi. Data represent mean ± SEM. *n* = 3 biological replicates analyzing ≥50 PVs for each condition per biological replicate, except for SCARB1. *P-*values display unpaired *t*-test. **P < 0.01; ****P < 0.001. (**E and F**) Huh7 cells were reverse transfected with the nontargeting siRNA Allstar (1 siRNA, 25 and 50 nM final), siRNAs targeting *SPHK1* or *CERS3* (2 siRNAs total, 12.5 nM each, 25 nM final), or pooled siRNAs targeting *SPHK1* and *CERS3* (4 siRNAs total, 12.5 nM each, 50 nM final) for 48 h before infection with *P. berghei*-Luc. The relative (**E**) Huh7 viability and (**F**) *P. berghei* load were assessed at 48 hpi. Data represent mean ± SEM. *n* = 4 biological replicates. *P*-values display unpaired *t*-test. ns = non-significant; **P < 0.01; ***P < 0.005; ****P < 0.001.

To identify host genes with the highest confidence of supporting the *Plasmodium* liver stage, we analyzed the overlap in the proposed targets from the small molecule and siRNA knockdown studies. This highlighted two proteins involved in the host sphingolipid salvage pathway, SPHK1 and CERS3, as critical to the liver stage (Fig. 6A). To understand their role in liver stage infection, we first assessed whether SPHK1 and CERS3 are involved in *P. berghei* invasion. Huh7 cells were treated with siSPHK1, siCERS3, and the positive control siSCARB1, before infection with *P. berghei.* At 3 hpi, cells were fixed, stained with anti-UIS4, and the intracellular infection rate was assessed by flow cytometry. Gene depletion of *SCARB1* led to a 58% ± 2.8% decrease in infected cells (*P* < 0.0001) when compared to Allstar. In contrast, we observed no change in the invasion rate for siSPHK1 and siCERS3 (Fig. 6B). At 48 hpi, siRNA knockdown of *SPHK1* and *CERS3* led to a >30% reduction in the number of infected cells and an approximate 50 µm^2^ decrease in PV size as measured with confocal immunofluorescence microscopy (Fig. 6C and D). These findings suggest that CERS3 and SPHK1 contribute to *P. berghei* liver stage development, and that their depletion enhanced parasite clearance, likely through increased susceptibility to host-mediated elimination.

Both SPHK1 and CERS3 function in the host salvage pathway, but CERS3 also has a role in the host *de novo* ceramide synthesis pathway (Fig. 5A). To probe the importance of the host salvage pathway vs *de novo* ceramide synthesis, we considered the effects of dual *SPHK1* and *CERS3* siRNA knockdown on parasite development. Huh7 cells were reverse transfected with 25 nM of two pooled siRNAs targeting *SPHK1* and *CERS3* (50 nM siRNA final), infected with *P. berghei*-Luc*,* and then assessed for cell viability and parasite load via the luciferase reporter at 48 hpi. Knockdown of both host genes had no effect on host cell viability (*P* = 0.16), but a 58% ± 3.4% reduction in *P. berghei* load (*P* < 0.0001) was observed (Fig. 6E and F). Confocal immunofluorescence microscopy analysis at 48 hpi also found a significant reduction in the intracellular infection rate and PV size (Fig. S15). Taken together, dual siRNA knockdown of *SPHK1* and *CERS3* did not have an additive effect when compared to each target individually, suggesting they act in the same pathway—consistent with their known role in the host salvage pathway for *P. berghei* development.

## DISCUSSION

During the liver stage, *Plasmodium* undergoes a dramatic transformation, expanding from a single sporozoite into tens of thousands of merozoites (2). This process requires a substantial supply of nutrients that the parasite must synthesize or acquire from its host. Some host lipids are known to be essential during the liver stage including cholesterol, phosphatidylcholine, and fatty acids (7, 13–15). Additionally, lipidomic and RNA sequencing studies have identified differentially regulated lipids and genes related to lipid synthesis during *Plasmodium* infection of hepatocytes (15, 46–49). These key studies have expanded our understanding of how different lipid species support the liver stage and motivated the testing of our lipid panel. While the presented work focused on sphingolipids, future efforts investigating the possible function of all lipids that increased [phosphatidylserine and PtdINs-(3,5)-P_2_] or decreased (1,2,3-tripalmitoyl-glycerol and phosphatidylglycerol) parasite load would be valuable for understanding their possible function during the liver stage. In this study, we found that sphingolipids were actively trafficked into the PV, supported *P. berghei* liver stage development, and increased nuclear replication. Furthermore, we discovered that host cell membrane sphingomyelin was required for parasite invasion. Finally, a combined small molecule and siRNA approach identified host genes involved in sphingolipid trafficking and metabolism that support liver stage *P. berghei* viability, hinting at druggable molecular pathways that could support lipid acquisition from the host.

The mechanism by which C16-ceramide promotes *Plasmodium* liver stage development remains unclear. Interestingly, while C16-ceramide is typically considered an antiproliferative sphingolipid in mammalian cells, we observed that it enhanced *Plasmodium* development in hepatocytes, suggesting a distinct parasite-specific response (50, 51). This could be a direct C16-ceramide effect, or the lipid could be modified after uptake through host or parasite-mediated mechanisms to induce proliferation. Of note, a similar proliferative effect has been observed in the related apicomplexan *Toxoplasma gondii*, where 5 µM C6-ceramide increased parasite replication by 130% (52). Here, we observed a similar 152% ± 16% increase in *P. berghei* load (*P* < 0.05) when cells were treated with 5 µM C6-ceramide, underscoring the complex roles these lipids play in apicomplexan biology.

*Plasmodium* is predicted to encode a sphingomyelin synthase, two sphingomyelinases, and a glucosyl-ceramide synthase-like enzyme, suggesting it has the enzymatic capacity to both use and modify host-derived sphingolipids (53–57). This has been demonstrated during the asexual blood stage where the parasite synthesizes sphingomyelin and glycosphingolipids from labeled precursors (57–59). Since liver stage *Plasmodium* cannot be separated from its host cell for lipidomic analysis, determining how the parasite uses scavenged sphingolipids will be a challenging yet important task. Future research could focus on identifying the active and essential *Plasmodium* sphingolipid biosynthetic genes used during the liver stage to clarify which sphingolipid species are required for parasite growth.

The role of sphingolipids has been investigated in stages of the *Plasmodium* lifecycle. Asexual blood stage *Plasmodium falciparum* has elevated sphingomyelin content that is essential for replication and the formation of the blood stage tubovesicular network (17, 18, 60–63). Fluorescence-based labeling studies have also shown that *P. falciparum* takes up C6-sphingomyelin, C6-ceramide, sphingosine-1-phosphate, sphingosine, and glycosphingolipids from the host cell and extracellular pools (16, 53, 64–66). During gametogenesis, nearly all sphingolipids are upregulated, particularly ceramides and complex sphingolipids (19, 61, 67). Sphingomyelin again plays a crucial role as it is specifically enriched in female gametocytes (19, 61, 67). Importantly, lipidomic studies of both the asexual and sexual blood stages have identified the ceramide precursor, dihydroceramide, providing compelling evidence that *Plasmodium* can synthesize sphingolipids *de novo* (19, 58, 67). Future work could resolve whether liver stage parasites complement lipid scavenging from its host cell with *de novo* sphingolipid synthesis. Additionally, it would be advantageous to perform studies throughout the *Plasmodium* lifecycle with C16- and C20-ceramide analogs that enable bioconjugation to fluorophores to investigate chain length specificity in live cell microscopy trafficking studies (68, 69).

To investigate how sphingolipids are trafficked to the PV, we focused on the host lipid transfer protein CERT1, which was previously identified in a screen as an essential host factor for *P. yoelii* liver stage viability, but not invasion (30). We observed an association of CERT1-V5 with the PVM at 48 hpi, and knockout of CERT1 significantly reduced *P. berghei* load and NBD C6-ceramide trafficking to the PV. Multiple *Chlamydia* species are also known to manipulate CERT1 for lipid acquisition. Specifically, the inclusion membrane protein IncD interacts with CERT1 to tether the bacterium to the host ER for ceramide trafficking (70–73). As *Plasmodium* also associates with the ER, it would be of interest to explore whether a similar protein to IncD exists in *Plasmodium* to facilitate CERT1 association to the PVM (12, 74).

Parasite load and ceramide trafficking to the PV were reduced in CERT1^KO^ cells, but not completely abolished. Furthermore, despite sphingolipid accumulation as early as 6 hpi, an impact on parasite load in CERT1^KO^ cells was not observed until 48 hpi. These observations suggest that CERT1-independent mechanisms exist to transport ceramide and other sphingolipids to the parasite. One possible mechanism could involve host vesicles of the endomembrane system. During the *T. gondii* lytic cycle, host Arf1-, Rab14-, Rab30-, and Rab43 vesicles are known to colocalize with exogenously tagged sphingolipids in the PV and are thought to aid in nutrient acquisition (52, 75, 76). In the case of *Plasmodium,* parasite viability is dependent on the expression of host Arfs, Rabs, and vesicle coat complexes, and these factors are known to associate with the PVM during infection (75, 77, 78). Future research is critical to elucidate whether host vesicles support sphingolipid trafficking to the PV. An alternative mechanism could involve other lipid transfer proteins. All analyzed lipid transfer proteins across our small molecule and siRNA studies emerged as putative regulators of the *Plasmodium* liver stage. These included CPTP (ceramide-1-phosphate transporter), PLKEHA8 (glucosylceramide transporter), and SPNS2 (sphingosine-1-phosphate transporter), suggesting that *Plasmodium* may rely on multiple host factors to access a diverse array of sphingolipids. Moreover, these host factors could create redundancies to ensure that essential lipid species are consistently available for parasite development. To further clarify these pathways, future experiments could include dual siRNA knockdowns in CERT1^KO^ cells, or a combination of knocking down multiple lipid transfer proteins. Comparing *Plasmodium* development in CERT1^KO^ cells vs CERT1 knock-in controls would also help identify independent and compensatory lipid acquisition mechanisms. Beyond lipid transport, our phenotypic investigation suggests that *CERS3* and *SPHK1* function in the same pathway to impact *Plasmodium* size and protection from clearance. This warrants further investigation as these enzymes both participate in the sphingolipid metabolic pathway yet have opposing effects on ceramide levels.

In contrast to the role of ceramide for parasite proliferation, sphingomyelin was found to be an important invasion factor. Cell membrane sphingomyelin has been linked to the successful invasion of various microorganisms including *Mycobacterium tuberculosis,* influenza A, West Nile virus, and hepatitis C virus (79–82). Similarly, we demonstrated that depleting cell membrane sphingomyelin with bSMase decreased *P. berghei* invasion. Previous studies during the *P. yoelii* liver stage identified an unknown secreted parasite factor that caused a redistribution of host lysosomes to the cell membrane prior to parasite invasion (83). Likewise, disrupting cell membrane lipid rafts with the small molecule inhibitor MβCD significantly decreased *P. yoelii* invasion (83). Given that both lysosomes and lipid rafts are rich in sphingomyelin, it is possible that they are critical for maintaining or elevating sphingomyelin levels on the cell membrane for invasion. With advancements in instrument sensitivities and methods to deconvolute parasite vs host processes, temporal and spatial lipidomic analyses could illuminate factors critical for productive invasion and early to mid-liver stage processes.

Targeting sphingolipid synthesis in *Plasmodium* has long been considered a promising strategy for combating malaria, given that ceramide homeostasis plays a crucial role in parasite viability (54, 84). Both insufficient and dysregulated sphingolipid levels can negatively impact the parasite throughout its lifecycle (60, 85, 86). Notably, two clinically relevant antimalarial drugs, artemisinin and mefloquine, activate parasite sphingomyelinase enzymes, leading to sphingomyelin depletion, reduced tubovesicular network formation, and parasite starvation during the asexual blood stage (60). Additionally, the host enzyme SPHK1 has previously been identified as an essential factor during the asexual blood stage by facilitating sphingosine trafficking and its conversion to sphingosine-1-phosphate in infected erythrocytes. Given its importance in both the liver and blood stages, SPHK1 represents a promising target for antimalarial drug development. Overall, continued research into the role of sphingolipids in both the host and parasite could reveal potent multi-stage inhibitors, offering new avenues for novel malaria therapies.

## MATERIALS AND METHODS

### Cell and parasite culture

Huh7 (Millipore Sigma, European Collection of Authenticated Cell Cultures) and HeLa (Duke Life Science Facility) cells were cultured in Dulbecco’s modified Eagle medium (DMEM, Gibco) supplemented with 10% heat-inactivated FBS (Millipore Sigma) and 1% antibiotic-antimycotic (Millipore Sigma) and maintained at 37°C with 5% CO_2_. *Anopheles stephensi* mosquitos infected with *P. berghei* ANKA (*P. berghei-*Luc) stably expressing luciferase (Luc) and *P. berghei* NK65 (*P. berghei*-RedStar) stably expressing RedStar (BEI Resources, NIAID, NIH: *Plasmodium berghei*, strain NK65 RedStar, MRA-905, contributed by Ute Frevert) were purchased from the SporoCore at the University of Georgia, Athens. *P. berghei* sporozoites were harvested from freshly dissected salivary glands immediately before experiments.

### Exogenous lipid screen and sphingolipid studies

Lipids used in the primary screen were selected based on their commercial availability and characterization in the literature. Each lipid was tested for their solubility in each solvent at 5 mM. Unfortunately, no single solvent was compatible with every lipid, in agreement with the product information and safety data sheets that informed their solubility. If multiple solvents were suitable, preference was given to H_2_O > EtOH > MeOH > CHCl_3_ > 3 mM NaOH. Compounds solubilized with chloroform and methanol were prepared fresh before each experiment. The commercial source and solvent selected for each lipid is described in Table S1.

Huh7, CERT1^WT^, and CERT1^KO^ cells (4,000/well) were seeded onto 384-well assay plates (Corning #3570) and allowed to recover overnight. For the exogenous lipid screen, cells were supplemented with 5 or 50 µM of each lipid or vehicle control (1%, vol/vol), and for the sphingolipid studies, cells were treated with C6-, C12-, C16-, and C20-ceramide, C16-sphingomyelin, or ethanol (1%, vol/vol) before infection with 4,000 *P. berghei-*Luc sporozoites per well. Cell viability was assessed at 24 or 48 hpi using CellTiter-Fluor (Promega) per the manufacturer’s instructions. Immediately afterward using the same microplate, parasite load was assessed with the parasite’s luciferase reporter using Bright-Glo (Promega) per the manufacturer’s instructions. The relative fluorescence and bioluminescence signal intensities were measured on an EnVision plate reader (PerkinElmer). Samples were evaluated with six technical replicates in 3 or 4 independent experiments. Data for each lipid were normalized to their respective solvent control.

To assess the effects of NBD C6-ceramide and NBD sphingosine on Huh7 viability, cells were treated with 5 µM of each lipid for 1 h before quantifying cell viability using CellTiter-Glo (Promega) per the manufacturer’s instructions. Samples were evaluated with six technical replicates in four independent experiments. Relative viability for NBD C6-ceramide was compared to those treated with H_2_O (1%, vol/vol), and NBD sphingosine was compared to ethanol (1%, vol/vol) treated cells.

### NBD sphingolipid acquisition

Huh7 (4,000/well) were seeded onto 384-well glass bottom microscopy plates (Cellvis-P38415HN) and allowed to recover overnight before infection with 4,000 *P. berghei*-RedStar sporozoites per well. Prior to indicated timepoints, nuclei were stained with Hoechst 33342 (1:2,000 in PBS) for 5 min at 37°C, washed three times with PBS, and supplemented with 5 µM NBD C6-ceramide complexed to BSA (solubilized in water, Invitrogen, cat # N226521) or NBD sphingosine (solubilized in ethanol, Cayman Chemical, cat # 25348) in FBS-free DMEM supplemented with 1% antibiotic-antimycotic. To inhibit parasite metabolism, *P. berghei-*infected Huh7 cells were treated with 1% DMSO or 10 nM atovaquone from 24 to 48 hpi or 24–36 hpi. Atovaquone was removed by washing cells three times with DMEM. A minimum of 10 parasites in four independent experiments were evaluated by live cell microscopy as described below.

### CERT1-V5 and CERT1-HA cloning and transfection

CERT1 was amplified from HeLa cDNA using primers in Table S2 and cloned into a pcDNA3 vector with a 19 amino acid linker to a V5 tag or a 2 amino acid linker to a HA tag by Gibson Assembly (New England Biolabs, Cat # E5520) per the manufacturer’s instructions. Successful insertion was confirmed by Sanger Sequencing.

For transfections, HeLa cells (3,000/well) were seeded onto 384-well glass bottom microscopy plates (Cellvis-P28415HN) and allowed to recover overnight. CERT1-V5 and CERT1-HA plasmids (75 ng/well) were transfected with Lipofectamine 3000 reagent (Invitrogen) according to the manufacturer’s protocol. CERT1-V5 expression in HeLa cells was confirmed by microscopy 72 h post transfection. For infections, 4,000 *P. berghei* sporozoites per well were added 24 h post transfection and fixed at 24 and 48 hpi. The evaluation of CERT1 association with the PVM is described below.

### Generation of CERT1 CRISPR knockout lines

The gRNA sequences for CERT1 were obtained from the Human CRISPR Knockout Pooled Library (GeCKO v2) and are listed in Table S3. gRNA1 targeted exon 2 and gRNA4 targeted exon 3 of CERT1 for Cas9 recruitment. The gRNAs were cloned into px459 (Addgene plasmid #62988) as previously described (87). Sanger sequencing was used to verify proper insertion of the gRNAs.

To generate a CERT1 knockout line, 300,000 HeLa cells were seeded onto a 6-well plate and allowed to recover overnight. Cells were then transfected with 2 mg each of px459_gRNA1 and px459_gRNA4 using lipofectamine 3000 (Invitrogen) according to the manufacturer’s instructions. To select for transfected cells, the media was supplemented with 2 mg/mL puromycin for 4 days. Clonal populations were obtained by single-cell diluting the surviving cells onto 96-well plates, which expanded for about 2 weeks with media changes every 3 days. Single-cell populations were screened for insertions and deletions in the CERT1 genome using Thermo Scientific Phire Tissue Direct PCR (Thermo Scientific, Cat # F-170S) per the manufacturer’s instructions with primers listed in Table S3. Candidate populations were then screened for CERT1 protein expression by western blot and RNA levels by qRT-PCR. CERT1^WT^ and CERT1^KO^ studies with lipid and ceramide treatments were completed as described above.

### Western blot

Confluent cells in a T75 flask were trypsinized, washed two times with PBS, resuspended in 100 µL of lysis buffer (25 mM HEPES, pH 7.4, 0.1% Triton-X100, 150 mM NaCl, 60 mM MgCl_2_, 1 mM DTT), and incubated on ice for 30 min. Cell lysates were clarified by centrifugation at 16,000 × *g* for 30 min at 4°C. Supernatant was collected and quantified by a Bradford protein assay (Thermo Scientific, cat# 23200). One hundred micrograms of total lysate was denatured with SDS at 95°C for 10 min and separated on a Novex 4%–20% tris-glycine gel. Proteins were transferred to a nitrocellulose membrane and then blocked for 1 h at RT with 3% BSA in PBS containing 0.2% Tween 20 (PBST). The blot was then probed with anti-CERT1 (Invitrogen cat # PA5-113546, 1:1,000) overnight at 4°C, washed with PBST, and incubated with anti-GAPDH (Invitrogen cat # MA5-15738; 1:1,000) for 1 h at RT. The membranes were again washed with PBST before incubation with Alexa Fluor conjugated antibodies (A21206 and A10037, 1:1,000) for 1 h at RT. Membranes were washed with PBST and imaged with a ChemiDoc MP system (BioRad).

### siRNA reverse transfection

For 384-well assays, Huh7 cells (3,000/well) were reverse transfected with 1:66 Lipofectamine RNAiMax (Invitrogen) to Opti-MEM (Gibco) with siRNAs at a final concentration of 25 nM for pooled (2/gene) and single siRNAs against a non-targeting scramble negative control (Allstar) and *SCARB1* as a positive control (Table S4). For dual siRNA knockdown studies, cells were reverse transfected with two pooled siRNAs against *SPHK1* and *CERS3* (4 siRNA total, 12.5 nM each, 50 nM final), compared to individual knockdown of *SPHK1* and *CERS3* (2 siRNA total, 12.5 nM each, 25 nM final), and normalized to the average Allstar value for both 25 nM and 50 nM final. No significant difference in cell viability or parasite load was observed for cells treated with 25 nM or 50 nM Allstar. For flow cytometry studies, Huh7 cells (200,000/well) were reverse transfected with siRNAs at a final concentration of 25 nM in 12-well plates.

RNA extraction was performed 48 h post reverse transfection to quantify gene expression at the time of *P. berghei* infection. Cells were subsequently infected with 4,000 or 100,000 *P. berghei*-Luc sporozoites per well for 384-well and 12-well assays, respectively.

Cell viability and parasite load were assessed as described above and normalized to Allstar at 48 hpi. Samples were evaluated in six technical replicates in 3–4 independent experiments. For analysis by flow cytometry, cells were fixed at 3 hpi and processed as described below.

### RNA extraction and qRT-PCR analysis

To validate *CERT1* disruption in CERT1^KO^ cells, confluent cells in a 6-well plate were trypsinized, washed with PBS, and lysed with RNA lysis buffer (Zymo). For siRNA knockdowns, six pooled wells of a 384-well plate were collected 48 h post-reverse transfection with RNA lysis buffer (Zymo).

Total RNA was extracted in CERT1^KO^ cells per the manufacturer’s protocol using the Quick-RNA MiniPrep. For siRNAs, RNA was extracted using the Quick-RNA MicroPrep kit (Zymo). Then, 0.5 µg of RNA was used for first-strand cDNA synthesis using random hexamers (Promega) and GoScript reverse transcriptase (Promega) per the manufacturer’s instruction. qRT-PCR analysis was performed with 2× universal SYBR green fast qPCR (Abclonal) and primers (Table S3) at a final volume of 5 µL in a 384-well plate and measured using a LightCycler 480 Instrument II (Roche Diagnostics). Cycle threshold (CT) values for each gene were normalized to the human housekeeping 18S rRNA CT values (CT[target] – CT[18S rRNA] = ΔCT). Data were normalized to WT cells or those treated with Allstar (ΔCT[experimental] − ΔCT[CTRL] = ΔΔCT) and the relative amount was calculated as 2^−ΔΔCT^. Samples were evaluated in triplicate in three independent experiments.

### Flow cytometry for the relative intracellular infection rate

For compounds, Huh7 cells (240,000/well) were seeded onto 12-well plates and allowed to recover overnight. For siRNA knockdowns, Huh7 cells (200,000/well) were reverse transfected with 25 nM siRNA onto 12-well plates for 48 h prior to infection as described above. Cells were infected with 100,000 *P. berghei*-Luc sporozoites per well and compounds of interest (1%, vol/vol) were added at the indicated concentrations. At 3 and 48 hpi, cells were detached with Accutase (Invitrogen, cat # 00-4555-56) and fixed with Cytofix Fixation Buffer (BD Biosciences, cat # 554655) at 4°C for 20 min. Cells were then washed with Perm/Wash Buffer (WB, BD Biosciences, cat # 554723) and blocked for 1 h at room temperature (RT) with 2% BSA in WB. Parasites were subsequently stained overnight at 4°C with goat polyclonal anti-UIS4 (antibodies.com, cat # A121573, 1:1,000) diluted in 2% BSA in WB. Cells were washed with WB before incubating with donkey anti-goat Alexa Fluor 647 (Invitrogen, cat # A-21447, 1:1,000) with 2% BSA in WB for 1 h at RT. Cells were subsequently washed with WB and resuspended in PBS containing 5 mM EDTA. Infected populations were identified using a BD-LSRII Cell Analyzer. Data were analyzed with FlowJo (TreeStar, version 10.1). The gating strategy used to identify infected cells is shown in Fig. S2 and S13A. Samples were evaluated in technical triplicates for three independent experiments.

### Sphingomyelin depletion by bacterial sphingomyelinase (bSMase)

Huh7 cells (4,000/well) were seeded onto 384-well glass bottom microscopy plates (Cellvis-P38415HN) or 384-well assay plates (Corning #3570) and allowed to recover overnight before infection with 4,000 *P. berghei*-Luc sporozoites per well. bSMase from *Bacillus cereus* (Millipore Sigma Cat # S7651) was resuspended in 25% glycerol in PBS (PBS/glycerol) to generate a 200 U/mL stock solution. At indicated time points, PBS/glycerol or bSMase (1%, vol/vol) were added to cells for the indicated time frames and then removed by washing cells three times with DMEM.

To assess bSMase activity on *P. berghei* sporozoites, parasites were pretreated with 2 U/mL bSMase or PBS/glycerol (1%, vol/vol) on ice for 3 h, RT for 20 min, and 37°C for 20 min. bSMase was removed from sporozoites by centrifuging at 1,500 × *g* for 5 min and washing two times with DMEM. Parasites were subsequently used to infect Huh7 cells. To ensure the bSMase enzyme remained viable after treatment, sporozoites pretreated with 2 U/mL bSMase or PBS/glycerol were subsequently used to infect Huh7 cells without washing. Samples were evaluated in six technical replicates in 3–4 independent experiments.

### Small molecule inhibitors

Myriocin (cat # 63150), fingolimod (cat # 11975), fumonisin B_1_ (cat # 62580), CAY10621 (cat # 13371), SLF1081851 hydrochloride (cat # 37150), desipramine hydrochloride (cat # 15304), GW 4869 hydrochloride hydrate (cat # 13127), and malabaricane C (cat # 29741) were purchased from Cayman Chemical. d,l-Threo-phenyl-2-palmitoylamino-3-morpholino-1-propanol (PPMP, cat # BML-SL215) and d,l-threo-1-phenyl-2- decanoylamino-3-morpholino-1-propanol (PDMP, cat # BML-SL210) were purchased from Enzo Biochem. Compounds were dissolved in DMSO.

Huh7 cells (4,000/well) were seeded onto 384-well assay plates (Corning #3570) and allowed to recover overnight. Cells were treated with 1 or 10 µM of each compound or DMSO (1%, vol/vol) before infection with 4,000 *P. berghei-*Luc sporozoites per well. At 48 hpi, cell viability and parasite load were assessed as described above. Samples were evaluated for six technical replicates in 3–4 independent experiments.

### Cell fixation and immunostaining

Uninfected and *P. berghei*-infected cells were fixed at the indicated times post infection for 15 min with 4% formaldehyde at RT or 37°C. Cells were washed three times with PBS before permeabilization with 0.1% Triton X-100 (Fisher Scientific) for 10 min at RT. Cells were washed again three times with PBS and blocked with 3% BSA and 0.05% Tween-20 (Millipore Sigma) in PBS (blocking buffer) for 1 h at RT. Primary antibodies were diluted in blocking buffer and incubated for 1 h at RT or overnight at 4°C. Primary antibodies included goat polyclonal anti-UIS4 (1 h RT, antibodies.com, cat # A121573, 1:400), mouse monoclonal anti-ceramide (overnight 4°C, Millipore Sigma, cat # C8104, 1:10), mouse monoclonal anti-V5 (1 h RT, Invitrogen, cat # R96025, 1:700), mouse monoclonal anti-HA (1 h RT, Santa Cruz, cat # SC-7392, 1:400), rabbit monoclonal anti-GM130 (overnight 4°C, Abcam, cat #AB52649, 1:200), and rabbit polyclonal anti-calnexin (overnight 4°C, Proteintech, cat # 10427-2-AP, 1:500). Cells were washed three times with PBS and incubated with Alexa Fluor-conjugated secondary antibodies (1:400) in blocking buffer for 1 h at RT (Invitrogen, cat #s A11055, A10037, A21206). Cells were washed three times before incubation with Hoechst 33342 (1:50,000 in PBS) for 10 min at RT and then washed three additional times with PBS.

### Confocal microscopy

Fixed and live cells were viewed on a Zeiss Airyscan 880 inverted confocal microscope with a Märzhäuser linearly encoded *x*,*y* stage and a 63× 1.4 NA oil-immersion plan Apochromat objective. Laser illumination was via Argon for 488 nm and diode for 405 nm and 561 nm. The fluorescence signal was collected with two photomultiplier tubes and one GaAsP detector in the following emission ranges for Hoechst 415–487 nm, for Alexa Fluor 488 and NBD 490–570 nm (GaAsP), and for Alexa Fluor 568 and RedStar 570–633 nm (GaAsP). Images were acquired sequentially by line scanning bidirectionally at 0.52 microseconds per pixel with line averaging of 4 and a size of 0.044 μm × 0.044 µm with pinhole calculated to be 1 airy unit for green using Zeiss Zen software (version 2.3). Files were saved as Carl Zeiss Image files. Z-stacks were acquired with 200 nm intervals with 15–25 slices per stack.

### Image analysis

FIJI was used to adjust image brightness and contrast, crop, and add scale bars (88). For PV size of fixed samples, images at the middle focal plane of the vacuole were acquired. Then, the innermost signal of the PVM, excluding tubovesicular features such as membrane clusters, was traced and measured. A minimum of 50 PVs per biological replicate were analyzed. To quantify the PV size and mean NBD fluorescent value of live samples, the total *P. berghei* cytosolic area was thresholded to the RedStar signal and measured. Then, the mean gray value, including holes, was calculated for the green channel within the same area. A minimum of 10 PVs per biological replicate were analyzed. To quantify the ceramide content after bSMase treatment, the mean fluorescent value of the full focal plane was measured for 10 images in 3 independent experiments and compared to cells treated with 25% glycerol in PBS. To investigate CERT1-V5 association with the PV, a plot profile along a line was generated for each channel. To quantify the relative intracellular infection rate at 48 hpi by microscopy, the total number of parasites per well for six technical replicates (wells) was quantified and compared to the control for three biological replicates.

Imaris (version 9.9.1) was used to study the number of nuclei per PV. Briefly, a surface of the PVM was generated by the UIS4 signal, and all signal outside of the surface was masked. Then, the Hoechst signal was converted into spots, threshold, and quantified for 40 parasites in 3 independent experiments.

### Data analysis

Data were analyzed with GraphPad Prism 10 using the indicated statistical tests described in the figure legends.

## SUPPLEMENTAL MATERIAL

Supplemental Material mbio.01675-25-s0001.pdf Figures S1 to S15; Tables S1 to S4.

ASM does not own the copyrights to Supplemental Material that may be linked to, or accessed through, an article. The authors have granted ASM a non-exclusive, world-wide license to publish the Supplemental Material files. Please contact the corresponding author directly for reuse.
