# Supplementary material for: Host sphingolipids support Plasmodium berghei liver stage development
Source: mBio. 2025 Jul 21;16(8):e01675-25. doi: 10.1128/mbio.01675-25 (PMC12345201; doi:10.1128/mbio.01675-25)
Supplement: Supplemental Material — Figures S1 to S15; Tables S1 to S4. [file mbio.01675-25-s0001.pdf]

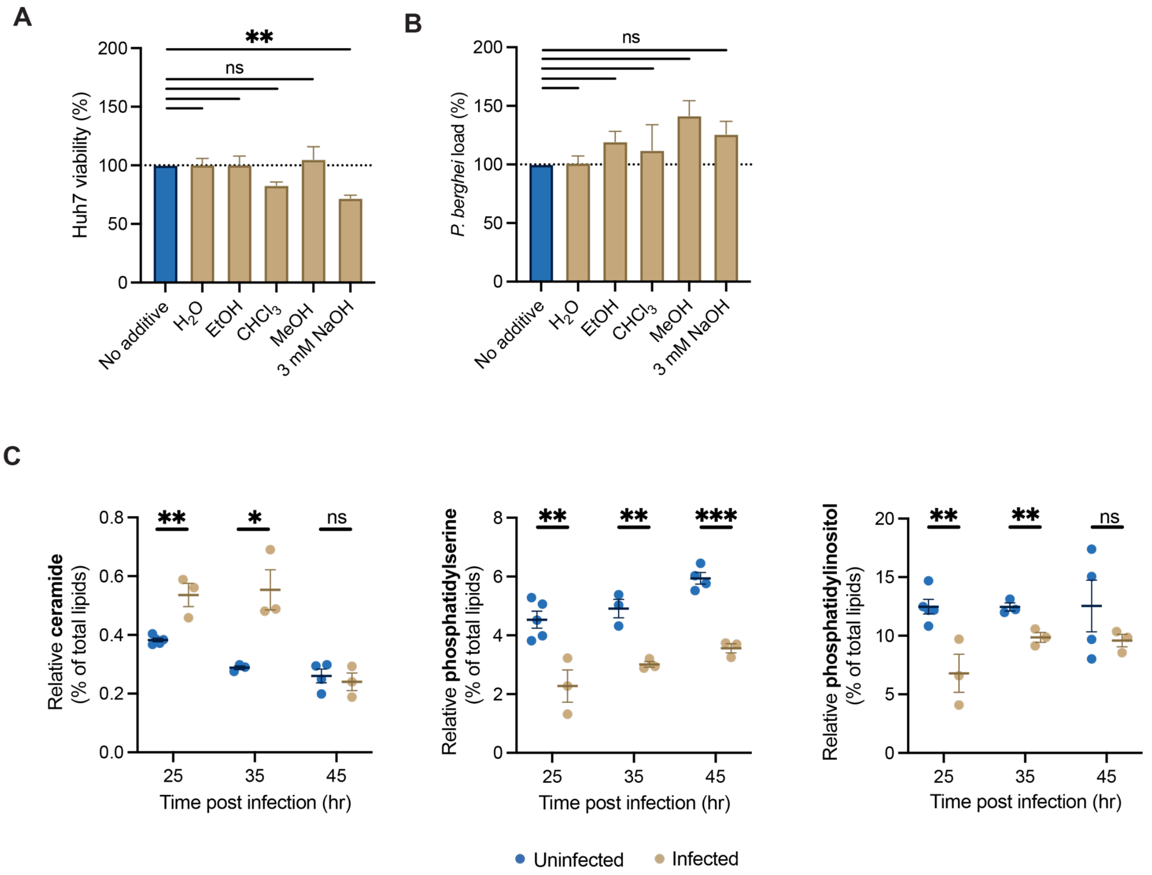

Supplemental Figure 1: Rationale for selecting solvents and lipids for analysis. (**A and B**) Huh7 cells infected with *P. berghei*-Luc were treated at the time of infection with water, ethanol, chloroform, or methanol at 1% v/v or 3 mM sodium hydroxide. At 48 hpi, the relative (**A**) Huh7 viability and (**B**) *P. berghei* load were assessed and compared to cells with no additive. Data represents mean ± SEM. n = 4 biological replicates. *P*-values display one-way ANOVA with Dunnett's multiple comparison test for each condition compared to the no additive control. ns = non-significant; ***P* < 0.01. (**C**) Quantitative shotgun lipidomics reported by Itoe and colleagues was used to determine the relative abundance of ceramide, phosphatidylcholine, and phosphatidylinositol in uninfected and *P. berghei*-infected Huh7 cells at 25, 35, and 45 hpi, (*Cell Host Microbe,* 2014). Data represents mean ± SEM. n = 3-5 biological replicates. *P*-values display unpaired t-test. ns = non-significant; **P* < 0.05; ***P* < 0.01; ****P* <0.005.

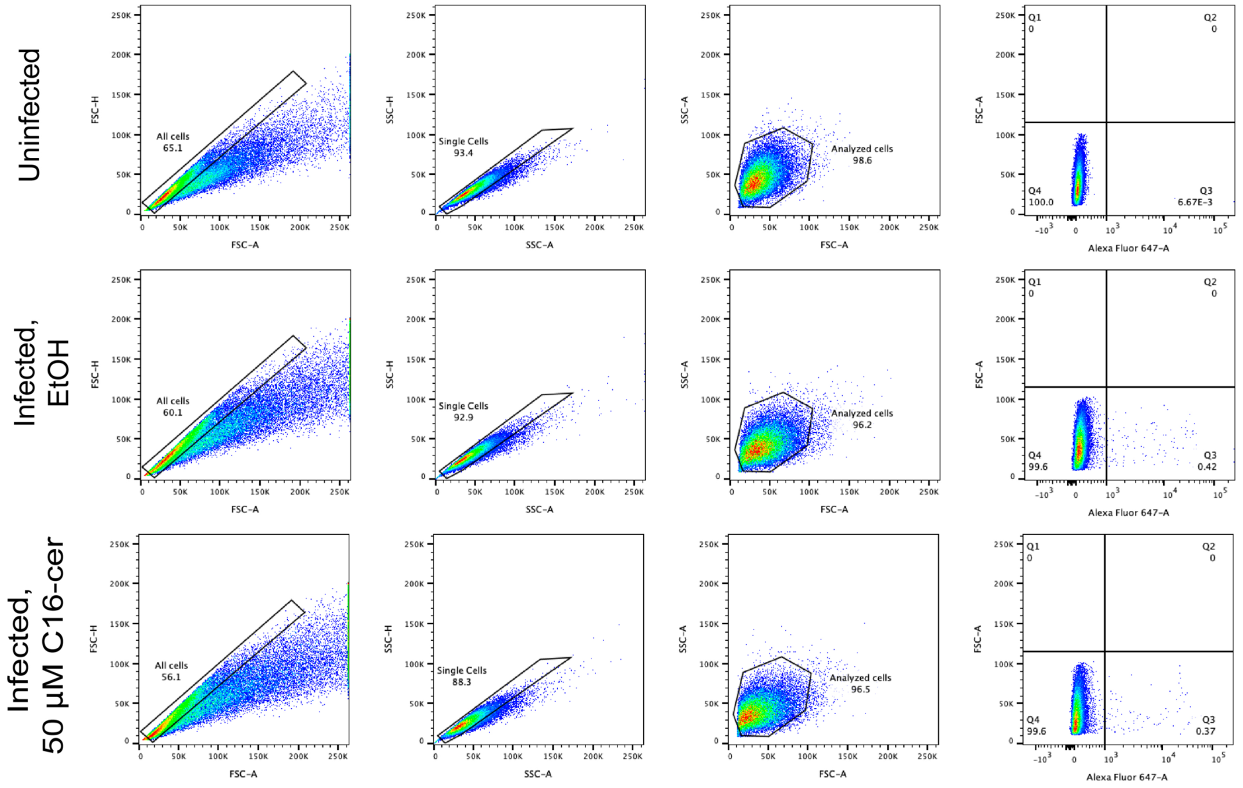

Supplemental figure 2: Flow cytometry gating strategy for *P. berghei* intracellular infection rate. Uninfected and *P. berghei*-Luc infected Huh7 cells were treated with 1% ethanol (CTRL) or 50 μM C16-ceramide at the time of infection. At 48 hpi, cells were fixed, permeabilized, stained for anti-UIS4, and conjugated to Alexa Fluor™ 647. Data represents example gating scheme for the identification of uninfected (Q4) and *P. berghei*-infected (Q3) cells. Data relates to Figure 1E.

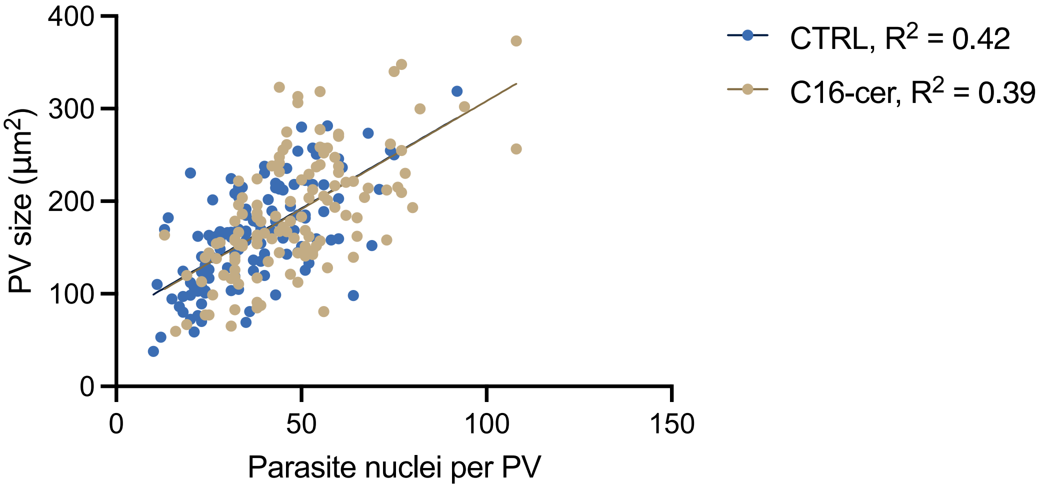

Supplemental figure 3: Relationship between PV size and parasite nuclei count. Huh7 cells infected with *P. berghei*-Luc were treated with 1% ethanol (CTRL, blue) or 50 µM C16-ceramide (brown) from 0-48 hpi. Z-stacks of the PVMs after UIS4 staining were acquired. FIJI was used to measure the PV size on the middle focal plane and Imaris was used to assign the number of nuclei per PV. n = 3 biological replicates analyzing ≥ 40 PVs for each condition per biological replicate. Data relates to Figure 1H.

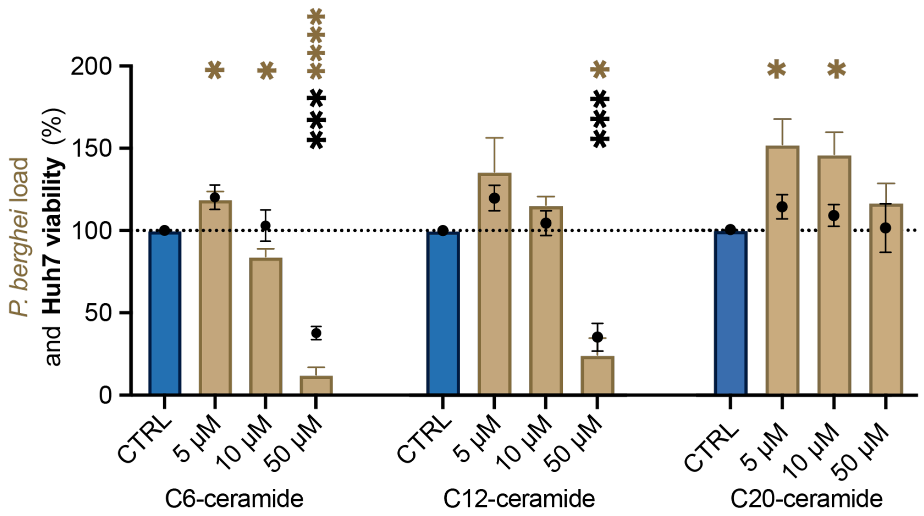

Supplemental Figure 4: Long chain ceramides promote *P. berghei* development. Huh7 cells were treated with increasing concentrations of C6-, C12-, and C20-ceramide at the time of *P. berghei*-Luc infection. The relative Huh7 viability (dots) and *P. berghei* load (bars) were assessed at 48 hpi and normalized to cells treated with 1% ethanol (CTRL). Data represents mean ± SEM. n = 3-4 biological replicates. *P*-values display one-way ANOVA with Dunnett's multiple comparison test for each condition to the CTRL. *$P < 0.05$; ***$P < 0.005$; ****$P < 0.001$.

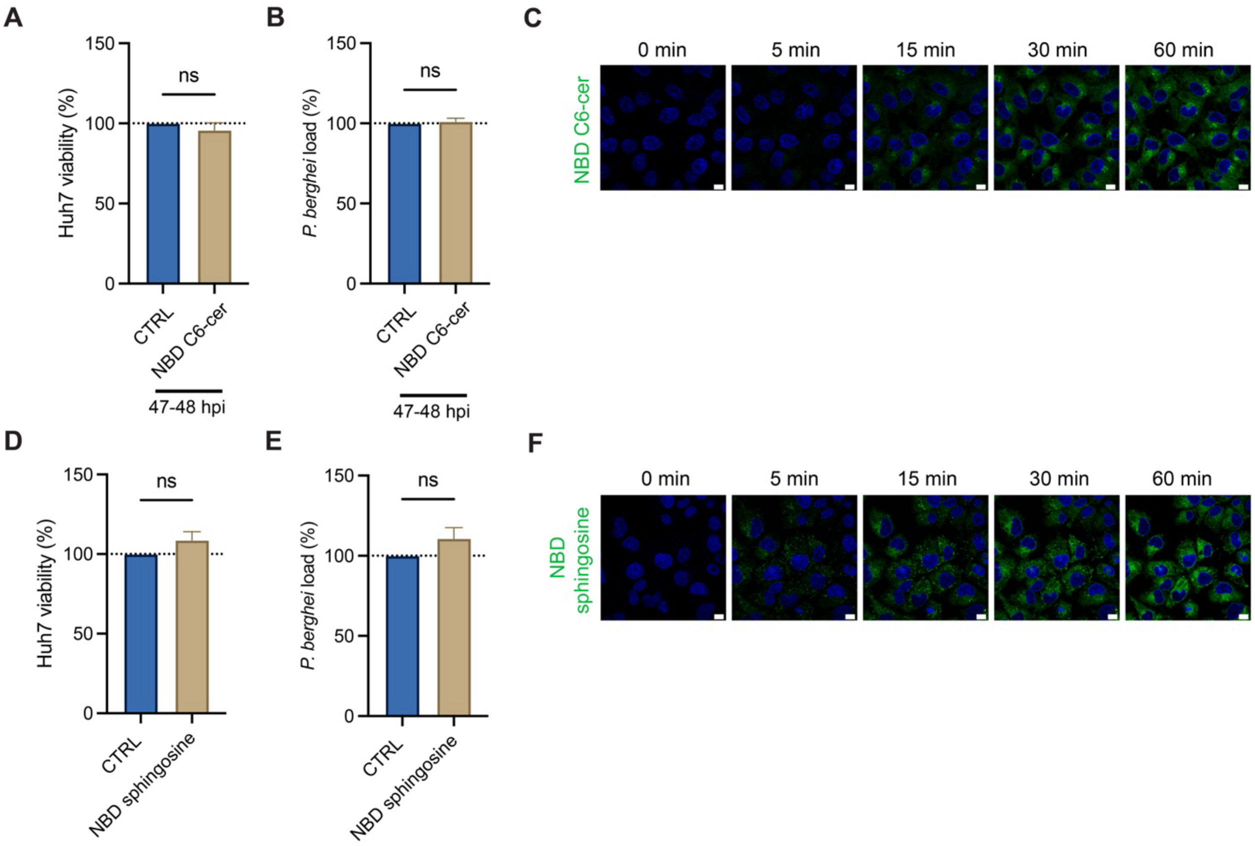

Supplemental Figure 5: Acquisition of exogenous NBD labeled sphingolipids. Uninfected and *P. berghei*-Luc-infected Huh7 cells were probed with 5 μM NBD C6-ceramide or NBD sphingosine from 47-48 hpi. (**A and D**) The relative Huh7 viability in uninfected cells (**B and E**) parasite load in *P. berghei*-Luc-infected cell is shown. Data represents mean ± SEM. n = 4 biological replicates. *P*-values display t-test. ns = non-significant. (**C and F**) Live confocal fluorescent images of Huh7 cells before and after the addition of 5 μM NBD C6-ceramide or NBD sphingosine (green) at the indicated times. Nuclei were stained with Hoechst (blue). Scale bars are 10 μM.

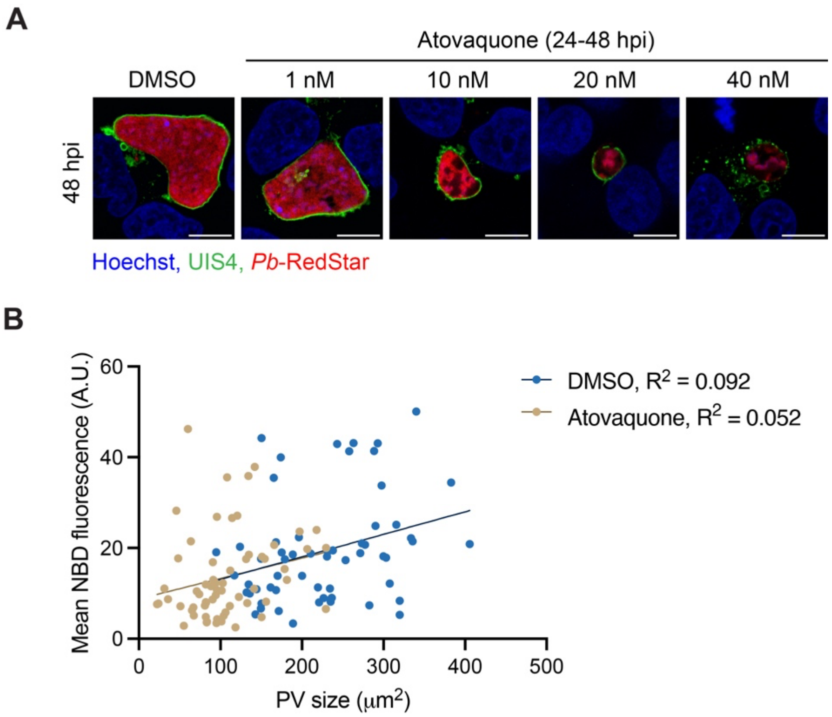

Supplemental Figure 6: Rationale for atovaquone treatment conditions and implementation. (**A**) Huh7 cells were infected with *P. berghei*-RedStar (red) and treated with 1% DMSO or varying concentration of atovaquone from 24-48 hpi. Representative confocal immunofluorescent images after atovaquone treatment demonstrates loss of the PVM integrity (UIS4, green) at 20 and 40 nM. Nuclei were stained with Hoechst (blue). Scale bars are 10 μm. (**B**) Huh7 cells infected with *P. berghei*-RedStar were treated with 1% DMSO or 10 nM atovaquone from 24-48 hpi and then supplemented with 5 μM NBD C6-ceramide for 1 hour prior to live cell microscopy. The relationship between parasite PV size and mean NBD fluorescence value inside the PV for DMSO (blue circles) and atovaquone (brown circles) treated parasites is shown. n = 4 biological replicates analyzing ≥ 15 PVs for each condition per biological replicate. Linear correlation between PV size and mean NBD fluorescence value is not observed for either condition. Data relates to Figure 2E and 2F.

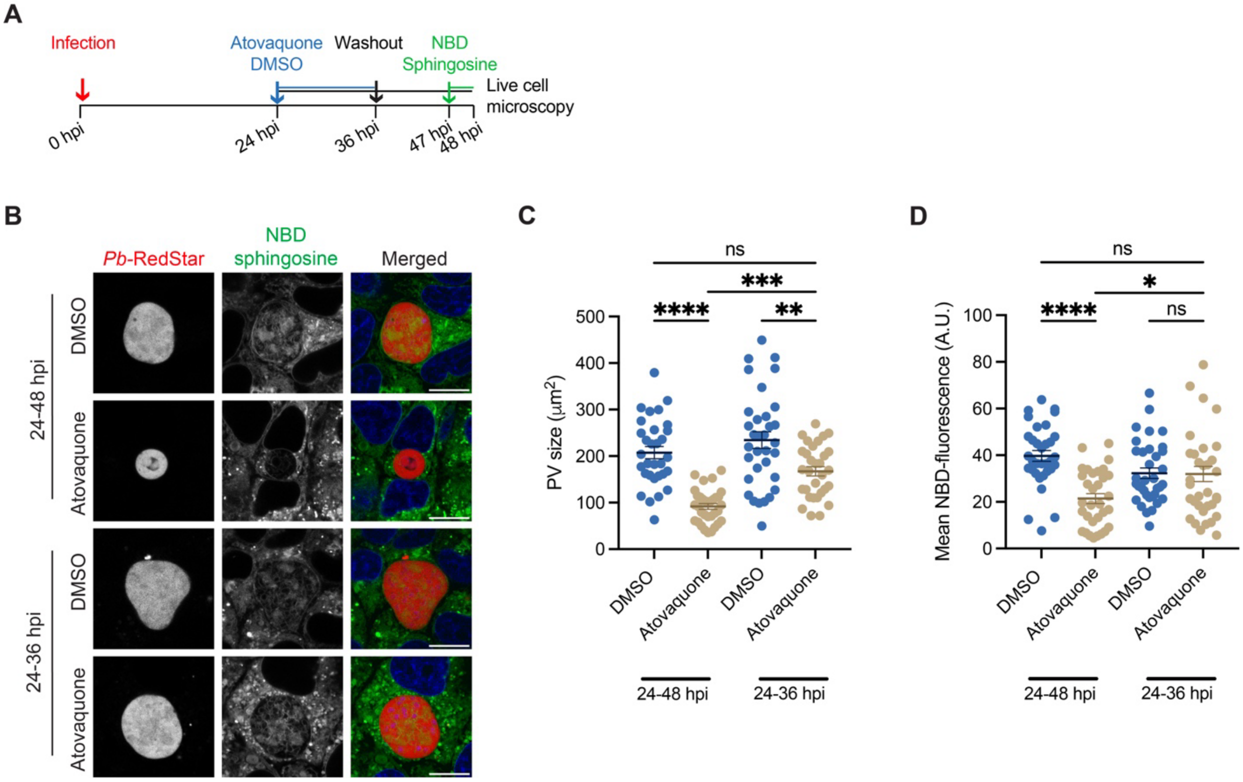

Supplemental Figure 7: *P. berghei* actively recruits NBD sphingosine to PV. (**A**) Schematic representation of NBD sphingosine uptake experiments. Huh7 cells infected with *P. berghei*-RedStar were treated with 1% DMSO or 10 nM atovaquone from 24-48 hpi or 24-36 hpi and then washed to remove the inhibitor. Cells were probed with 5 µM NBD sphingosine from 47-48 hpi before live cell microscopy (**B**) Representative live confocal microscopy images of NBD sphingosine (green) acquisition by *P. berghei*-RedStar (red). Nuclei were stained with Hoechst (blue). Scale bars are 10 µm. (**C**) The PV size was assessed at 48 hpi by measuring the RedStar area by microscopy. Data represents mean ± SEM. n = 3 biological replicates analyzing ≥ 10 PVs for each condition per biological replicate. *P*-values display unpaired t-test. ns = non-significant; ***P* < 0.01; ****P* < 0.005; *****P* < 0.001. (**D**) Quantification of the mean NBD fluorescence value inside the PV. Data represents mean ± SEM. n = 3 biological replicates analyzing ≥ 10 PVs for

each condition per biological replicate. P-values display unpaired t-test. ns = non-significant; *$P < 0.05$; ****$P < 0.001$.

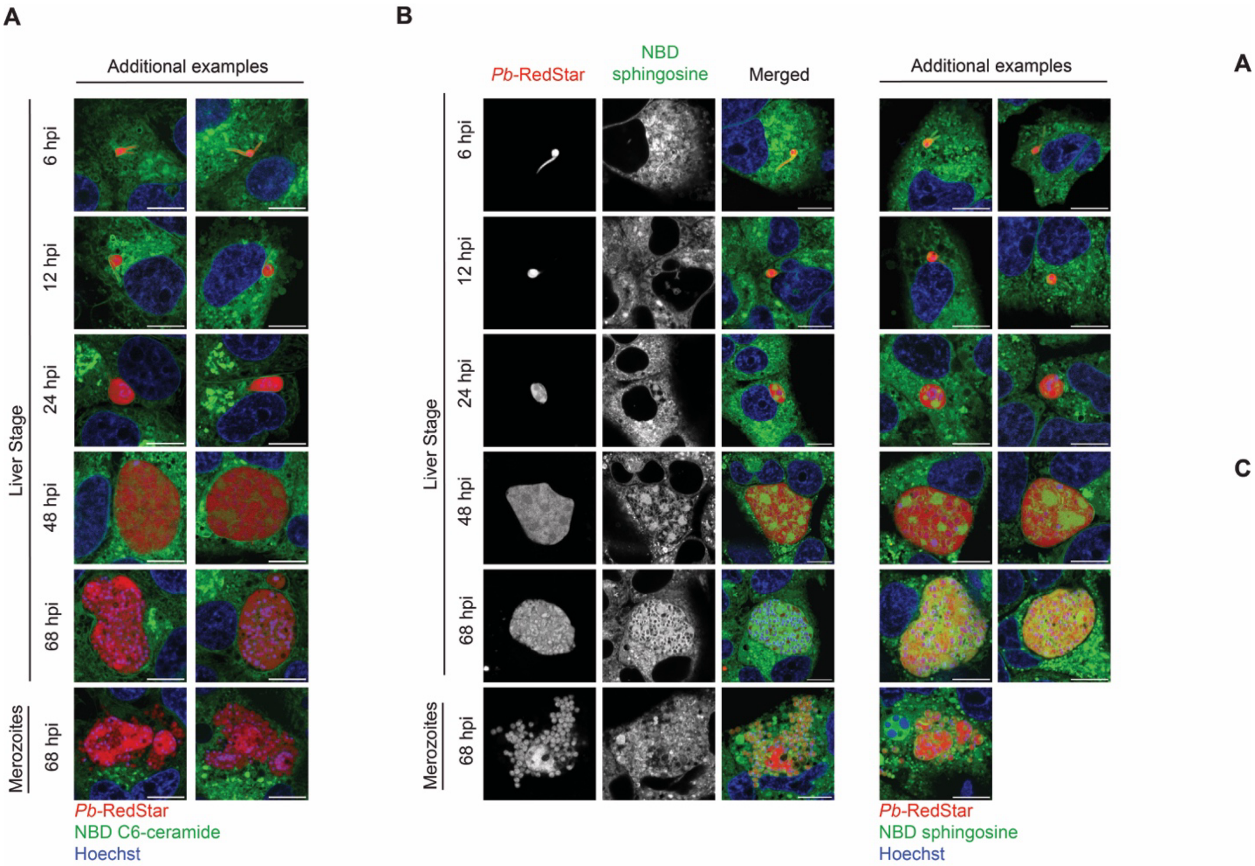

Supplemental Figure 8: Accumulation of NBD C6-ceramide and NBD sphingosine in the *P. berghei* PV. Live confocal microscopy images of *P. berghei*-RedStar (red)-infected Huh7 cells treated with 5 μM (**A**) NBD C6-ceramide (green) or (**B**) NBD sphingosine (green) for 1 hour prior to microscopy at the indicated times post infection. Nuclei were stained with Hoechst (blue). Liver stage parasites and merozoites are shown. Scale bars are 10 μm. Images in (**A**) relate to Figure 2G.

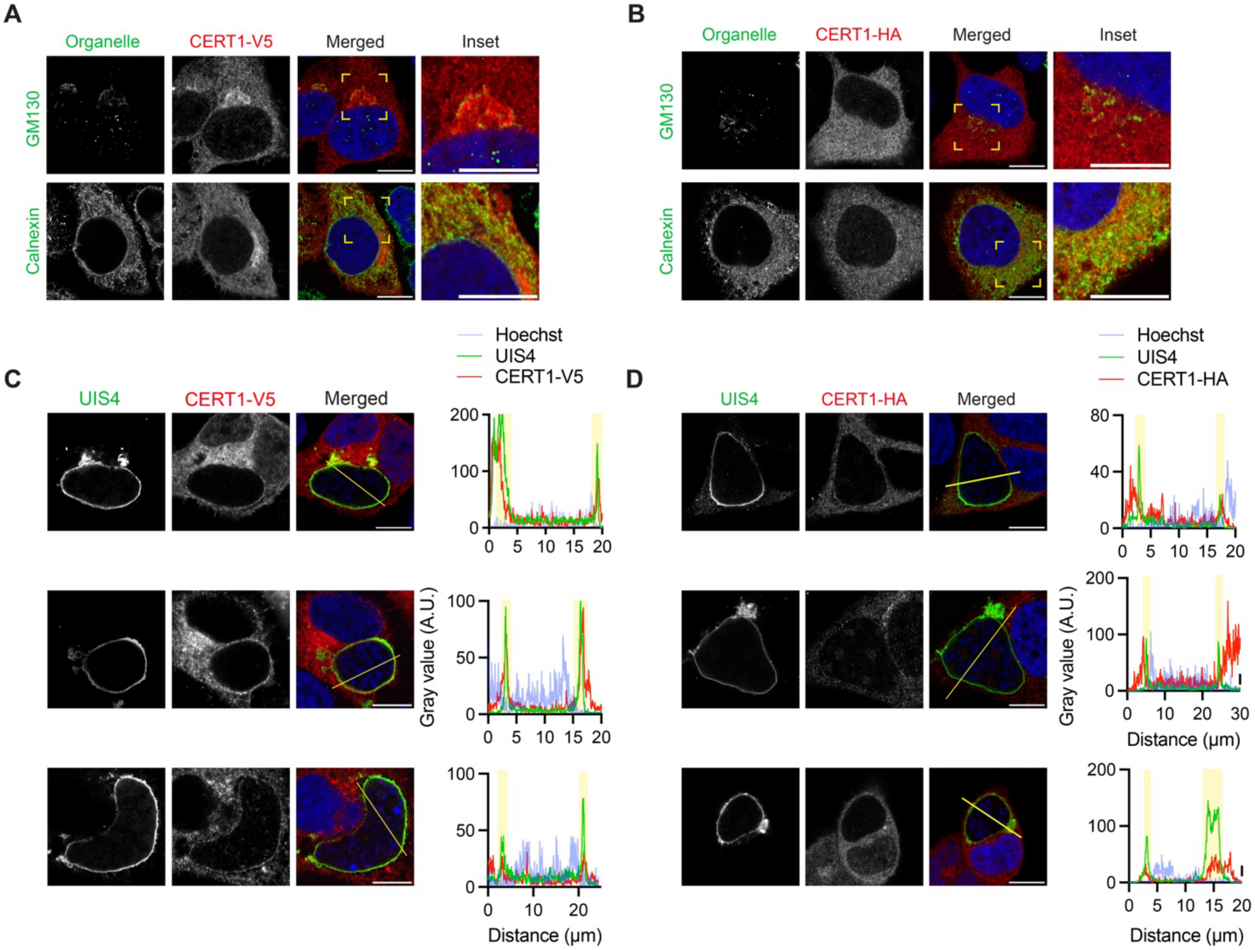

Supplemental Figure 9: CERT1-V5 and CERT1-HA localization at 48 hpi. (**A and B**). HeLa cells overexpressing (**A**) CERT1-V5 (red) and (**B**) CERT1-HA (red) were stained with the cis-Golgi marker GM130 (green) or the ER marker calnexin (green). Nuclei were stained with Hoechst (blue). Insets (yellow boxes) are shown to visualize the association of CERT1 with organelle markers. (**C and D**) HeLa cells overexpressing (**C**) CERT1-V5 (red) and (**D**) CERT1-HA (red) were infected with *P. berghei* and fixed at 48 hpi. Cells were stained with anti-UIS4 (green), and nuclei were stained with Hoechst (blue). Plot profiles of the gray value for each fluorophore across the yellow line is shown. Scale bars are 10 µm.

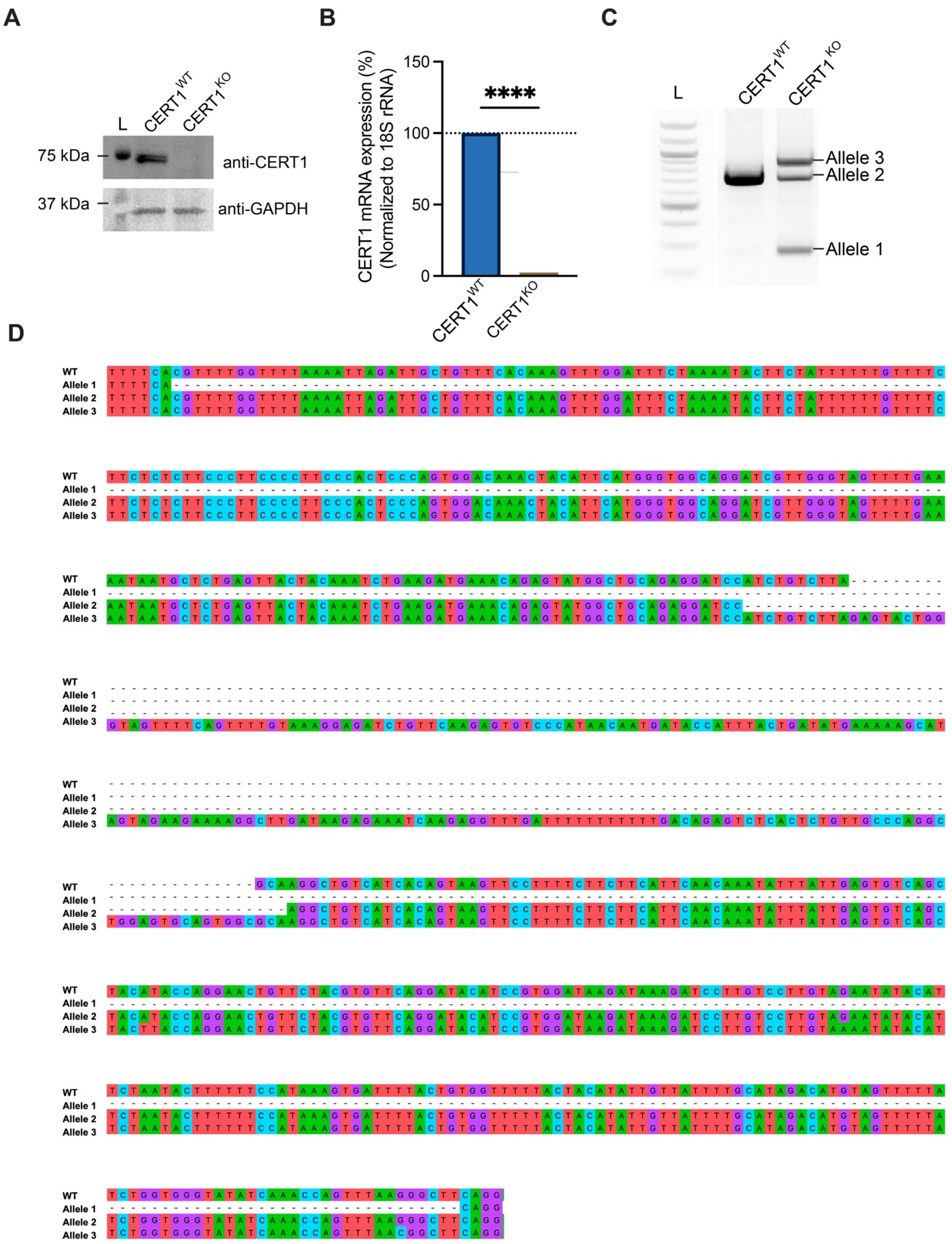

Supplemental Figure 10: Validation of CERT1 CRISPR disruption line. (**A**) Representative western blot image comparing CERT1 protein expression in CERT1^{WT} and CERT1^{KO} HeLa cells. GAPDH was used as a loading control. (**B**) The relative *CERT1* mRNA expression was assessed for CERT1^{WT} and CERT1^{KO} HeLa cells by qRT-PCR. Data were normalized to 18S. Data represents mean ± SEM. n = 3 biological replicates. *P*-value display unpaired t-test. ****$P < 0.001$. (**C and D**) Exon 2 of CERT1 was amplified from CERT1^{WT} and CERT1^{KO} genomic DNA resulting

in three distinct bands for CERT1^{KO} cells. Sanger sequencing identified three mutant alleles: a 481 bp indel (allele 1), a 13 bp deletion (allele 2), and a 181 bp indel (allele 3).

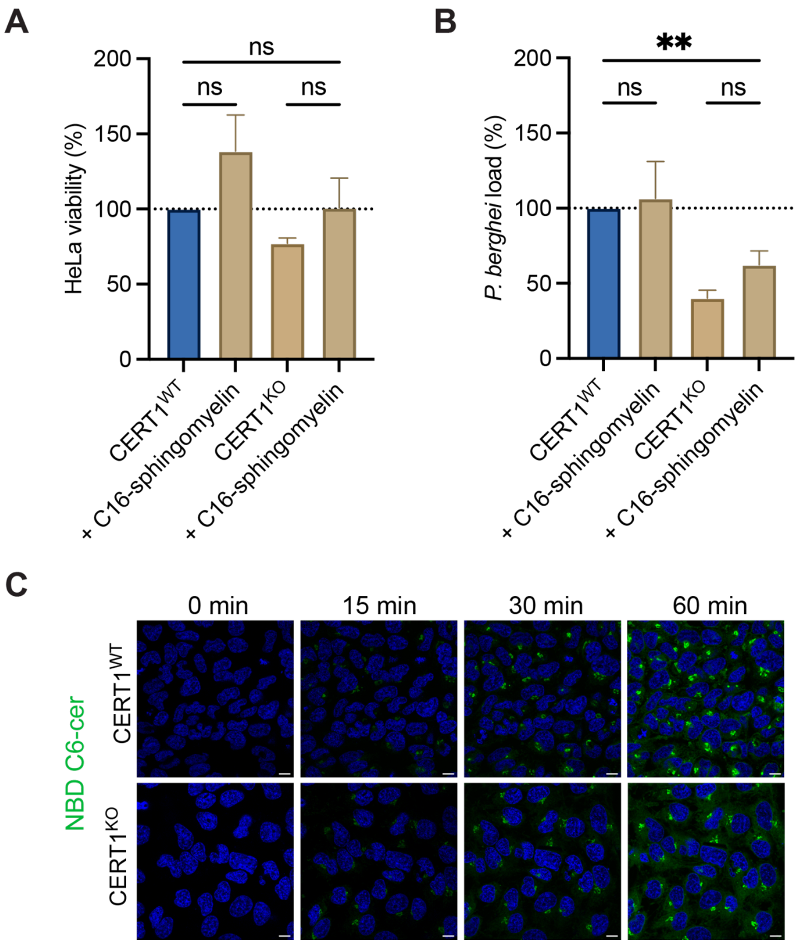

Supplemental Figure 11: Ceramide acquisition by CERT1^{WT} and CERT1^{KO} cells. (**A and B**) CERT1^{WT} and CERT1^{KO} cells were infected with *P. berghei*-Luc and treated with 1% ethanol (CTRL) or 50 μM C16-sphingomyelin. (**A**) HeLa viability and (**B**) parasite load were assessed at 48 hpi and normalized to the control. Data represents mean ± SEM. n = 4 biological replicates. *P*-values display unpaired t-test. ns = non-significant; $^{**}P < 0.01$. (**C**) Live confocal microscopy images of CERT1^{WT} and CERT1^{KO} cells before and after the addition of 5 μM NBD C6-ceramide (green) at the indicated times. Nuclei were stained with Hoechst (blue). Scale bars are 10 μm.

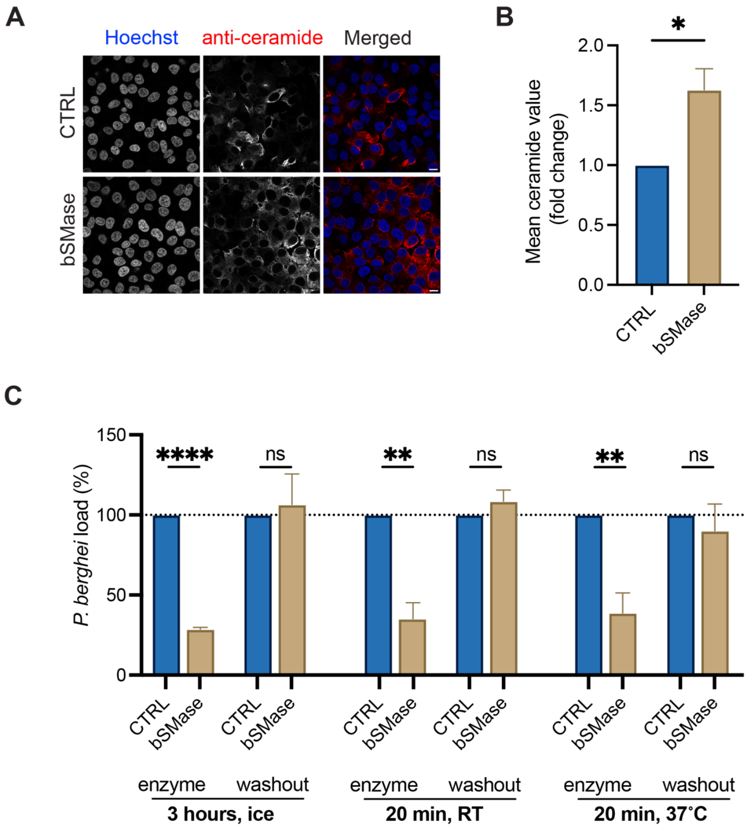

Supplemental Figure 12: bSMase acts on host cell sphingomyelin and does not impair *P. berghei* sporozoite viability. (**A and B**) Uninfected Huh7 cells were treated with 25% glycerol in PBS (CTRL) or 0.5 U/mL bSMase for 2 hours at 37°C before fixation. (**A**) Nonpermeabilized cells were stained with anti-ceramide (red) and nuclei were stained with Hoechst (blue). Scale bars are 10 μm. (**B**) The mean ceramide value of the full focal plan was measured for 10 images per biological replicate and normalized to the control. Data represents the mean fold change ± SEM. n = 3 biological replicates. *P*-values display unpaired t-test *$P < 0.05$. (**C**) *P. berghei*-Luc sporozoites were treated with 25% glycerol in PBS (CTRL) or 2.0 U/mL bSMase and incubated for 3 hours on ice, 20 minutes at room temperature (RT), or 20 minutes at 37°C. The pretreated parasites were either used directly for infection (enzyme) or washed two times with DMEM to remove bSMase before infection (washout). The relative *P. berghei* load was assessed at 48 hpi and normalized to the control. Data represents mean ± SEM. n = 3-4 biological replicates. *P*-values display unpaired t-test. ns = non-significant; **$P < 0.01$; ****$P < 0.001$.

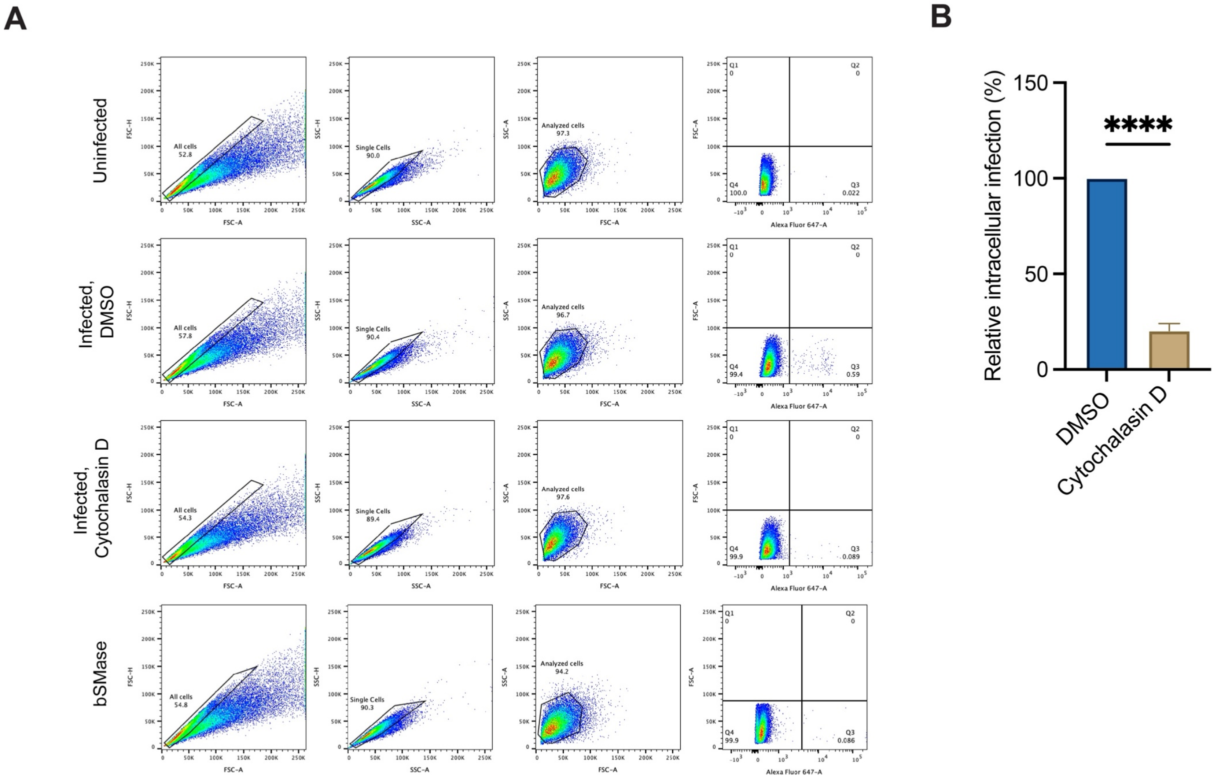

Supplemental Figure 13: Cytochalasin D inhibits *P. berghei* invasion assessed by flow cytometry. (**A-B**) Uninfected and *P. berghei*-Luc-infected Huh7 cells treated with 1% DMSO (CTRL), or 0.5 μM cytochalasin D at the time of infection. At 3 hpi, cells were fixed, permeabilized, stained for anti-UIS4, and conjugated to Alexa Fluor™ 647. (**A**) Data represents example gating for uninfected and infected samples for the identification of *P. berghei*-infected (Q3) and uninfected (Q4) cells. (**B**) The relative intracellular infection rate was assessed at 3 hpi. Data was normalized to the DMSO control. Data represents mean ± SEM. n = 3 biological replicates. *P*-value display unpaired t-test. ****$P < 0.001$.

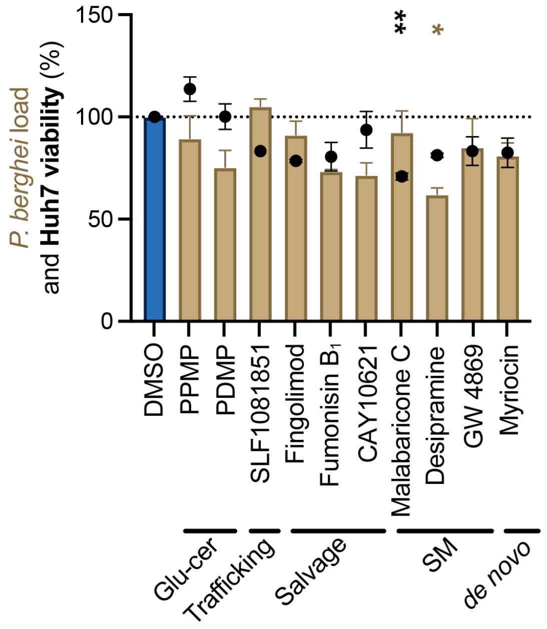

Supplemental figure 14: Anti-*Plasmodium* activity of lipid pathway inhibitors at 1 μM. *P. berghei*-Luc infected Huh7 cells were treated with ten small molecules known to target host ceramide metabolism at a final concentration of 1 μM at the time of infection. Huh7 viability (black circles) and *P. berghei* load (bars) were assessed at 48 hpi and normalized to the DMSO (1%) control. Data represents mean ± SEM. n = 3 biological replicates. *P*-values display one-way ANOVA with Dunnett's multiple comparison test for each condition compared to DMSO (black dashed line). *$P < 0.05$; **$P < 0.01$.

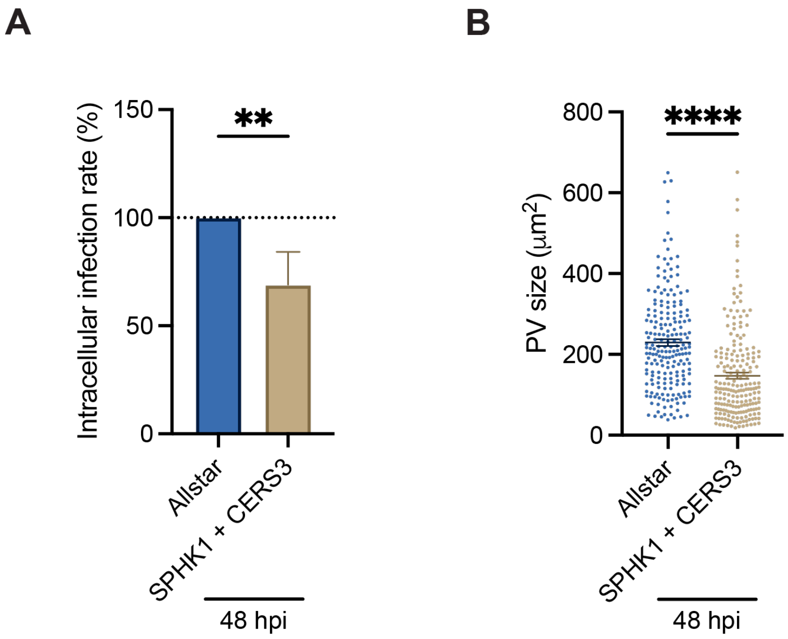

Supplemental figure 15: Dual siRNA knockdown reduces parasite infection rate and PV size. Huh7 cells were reverse transfected with the nontargeting siRNA Allstar (50 nM final), or pooled siRNAs targeting SPHK1 and CERS3 (4 siRNAs total, 12.5 nM each, 50 nM final) for 48 hours before infection with P. berghei. At 48 hpi, the (A) intracellular infection rate and (B) PV size was measured by confocal immunofluorescence microscopy. Data represents mean ± SEM. n = 4 biological replicates analyzing ≥ 50 PVs for each condition per biological replicate. P-values display unpaired t-test. **P < 0.01; ****P < 0.001.

Supplemental Table 1: Lipids and respective solvents

Name / Source	Type	Solvent	μM	Mean Huh7 viability	Mean *P. berghei* load
**1,2,3-tripalmitoyl-glycerol**<br>Cayman Chemical, cat # 23334	Neutral lipid	Chloroform	5	86.8	69.4
			50	78.5	70.3
**Cholesteryl palmitate**<br>Cayman Chemical, cat # 26473	Neutral lipid	Chloroform	5	80.8	76.4
			50	60.2	74.9
**1,2-dihexanoyl-*sn*-glycerol**<br>Cayman Chemical, cat # 10008646	Neutral lipid	Ethanol	5	131.7	113.1
			50	140.0	116.9
**1,2-Dipalmitoyl-*sn*-glycero-3-phosphocholine (DPPC)**<br>Echelon, cat # L-1116	Phospholipid	Chloroform	5	56.8	29.2
			50	29.9	19.2
**1,2-Dipalmitoyl-*sn*-glycero-3-phosphoserine (DPPS)**<br>Echelon, cat # L-3116	Phospholipid	3 mM NaOH	5	114.8	119.1
			50	129.0	127.9
**1,2-Dipalmitoyl-*sn*-glycero-3-phosphoglycerol (DPPG)**<br>Echelon, cat # L-5116	Phospholipid	Methanol	5	99.8	119.4
			50	94.3	71.2
**PtdIns-(3,5)-P_2**<br>Cayman Chemical, cat # 10007763	Phospholipid	H_2O	5	81.0	130.0
			50	104.8	108.9
**C16-ceramide**<br>Cayman Chemical, cat # 10681	Sphingolipid	Ethanol	5	125.3	120.9
			50	118.0	126.5
**C16-sphingomyelin**<br>Cayman Chemical, cat # 10007946	Sphingolipid	Ethanol	5	135.2	103.6
			50	133.6	85.6
**Sphingosine-1-phosphate**<br>Cayman Chemical, cat # 62570	Sphingolipid	3 mM NaOH	5	132.6	79.2
			50	87.4	60.8
**Cholesterol**<br>Cayman Chemical, cat # 39088	Sterol	Ethanol	5	142.5	100.4
			50	140.7	104.1
**C6-ceramide**<br>Cayman Chemical, cat # 62525	Sphingolipid	Ethanol			
**C12-ceramide**<br>Cayman Chemical, cat # 22530	Sphingolipid	Ethanol			
**C20-ceramide**<br>Cayman Chemical, cat # 10724	Sphingolipid	Ethanol			

Supplemental Table 2: Primers used for qRT-PCR and PCR analysis.

Name		Sequence (5' → 3')	Application
18S rRNA	Forward	GGCCCTGTAATTGGAATGAGTC	qRT-PCR
	Reverse	CCAAGATCCAACTACGAGCTT	
SCARB1	Forward	TCTCTCCGTCTACTTCTTTGACG	qRT-PCR
	Reverse	AACTGGAAGGTGCGGTACTC	
CPTP	Forward	GATGACTCGGAGACAGGTTT	qRT-PCR
	Reverse	CCACGTCCTTGGAGATGAAT	
PLEKHA8	Forward	GTAGCCCAGGTTAGGAACTC	qRT-PCR
	Reverse	GCCCTTAAAGCTAACGCAAA	
SPNS2	Forward	CCAAGTTGTGCAGAAGACAG	qRT-PCR
	Reverse	CCCAGTTAGAAAACAGCAGC	
ACER2	Forward	CGAGGACAACTACACCATCG	qRT-PCR
	Reverse	ACGGATCCAATTCCCACTAC	
CERS2	Forward	CCGAGATGGACGTGTCTACG	qRT-PCR
	Reverse	GCCGGGACAAAAGCTCTACT	
CERS3	Forward	ACATTCCACAAGGCAACCATTG	qRT-PCR
	Reverse	CTCTTGATTCCGCCGACTCC	
SPHK1	Forward	GCGTCATGCATCTGTTCTAC	qRT-PCR
	Reverse	GCCTCGCTAACCATCAATTC	
SGMS2	Forward	TCGTCACTTCTGGTGGTATC	qRT-PCR
	Reverse	ACCACCATGCTCGAGATAAG	
SMPD1	Forward	GGCCCACATTTGGGAAAGTT	qRT-PCR
	Reverse	TTCACCGGATGATCTTGCCT	
SMPD2	Forward	TTTGCTGGAGGAGGTGTGGAG	qRT-PCR
	Reverse	AAGCTCCTGGATTGGATGTTTGG	
SMPD4	Forward	CCAGCAAAGGGTCCTGTGAA	qRT-PCR
	Reverse	CTGAAGTACGGACGTGGAGG	
SPTLC1	Forward	TACGAGGCTCCTGCTTACCA	qRT-PCR
	Reverse	CTTGGAGGGCCTGAAACGAT	

CERT1	Forward	GCTGTCATCACACCTCACGA	qRT-PCR
	Reverse	CAGGGACACCATTGAGCCAT	
CERT1	Forward	AGGAGAGGATGAAAGCATACGA	PCR, Sanger sequencing
	Reverse	AACTGTCCCAGCCTCAACAAA	
CERT1-V5 cloning	Insert Forward	CGCCAGTGTGCTGGAATTCATGTCGGATAATCAGAGCTG	Cloning
	Insert Reverse	CAGCTCTGATTATCCGACATGAATTCCAGCACACTGGCG	Cloning
	Backbone Forward	CAGGAAAGCCTATTTTGTTCGATGAATTCTGCAGTCGACG	Cloning
	Backbone Reverse	CGTCGACTGCAGAATTCATCGAACAAAATAGGCTTTCCTG	Cloning
CERT1-HA cloning	Insert Forward	CTCGGATCCGCCACCATGTCGGATAATCAGAGC	Cloning
	Insert Reverse	TGGTACATCGTATGGATACTCGAGGAACAAAATAGGCTTTCCTG	Cloning
	Backbone Forward	CAGGAAAGCCTATTTTGTTCCTCGAGTATCCATACGATGTACCAG	Cloning
	Backbone Reverse	GCTCTGATTATCCGACATGGTGGCGGATCCGAG	Cloning

Supplemental Table 3: gRNAs used for generating CERT1 mutant cells with CRISPR/Cas9.

**Gene Name**	**GeCKOv2 Library Name**	**Sequence**	**Target**
CERT1	HGLibB_10563	AGGATCCATCTGTCTTAGCA	Exon 2
CERT1	HGLibA_10574	ATCCATTGCTGTCTATGATC	Exon 3

Supplemental Table 4: siRNAs used for gene depletion.

Gene Name	siRNA target sequence	Qiagen Product ID, except where noted
Allstar	Proprietary	SI03650318
SCARB1	ACTGACCGGACGTGGGATT	Dhamacon D-010592-03
ACER2	CTGGCAGTAGCTATGCACTCA	SI04237800
ACER2	CAAGTTCTGGCCCAATGAGAA	SI04183074
CERS2	CATGGCCGTCATTGTGGATAA	SI04276671
CERS2	TGCGCTATAGGGTCACTTTAA	SI04296684
CERS3	CAGTTCGAAAGGTTACACCAA	SI03178987
CERS3	CTCGTGATGATTGTACACGAT	SI04341771
CPTP	TCGGTGGAGCAGGGACCCGAA	SI04312686
CPTP	CTCCAAGGACGTGGTCTCCAA	SI04286037
SGMS2	AACGATTAGAAAGATGAACAA	SI02758182
SGMS2	CACGAACACTACACTATCGAT	SI03059462
PLEKHA8	AAGGATATCCAGACAGCCCTA	SI05587526
PLEKHA8	ATGGCAGTCTGTGAAATTCAA	SI05587533
SMPD1	CCGCCTCATCTCTCTCAATAT	SI00011578
SMPD1	CTGCTGTGGGTAACCATGAAA	SI03096121
SMPD2	CCGCATTGACTACGTGCTTTA	SI02655114
SMPD2	AACATCCAATCCAGGAGCTTA	SI04337928
SMPD4	CCCACAGTGGTTTGCTAAGAA	SI04169704
SMPD4	ATGGGCTGCGAAGGTTTGAAA	SI05458082
SPHK1	CTGCCTATGTAAGGCCTTCTA	SI02660455
SPHK1	AAGGATGGGAAAGGTGTGTTT	SI02758840
SPNS2	CTGCACTTCTGCTGCAATCAA	SI04765929
SPNS2	CCCACACAACTTGCTGGGCAA	SI04765936
SPTLC1	CCGGAAGGAAGCGGCTAACTA	SI04952395
SPTLC1	GTGAAGCTAAATGGTGGCTAA	SI05124812
